# Supplementary material for: Development, deployment and evaluation of digitally enabled, remote, supported rehabilitation for people with long COVID-19 (Living With COVID-19 Recovery): protocol for a mixed-methods study
Source: BMJ Open. 2022 Feb 7;12(2):e057408. doi: 10.1136/bmjopen-2021-057408 (PMC8822541; doi:10.1136/bmjopen-2021-057408)
Supplement: Author's manuscript [file bmjopen-2021-057408.draft_revisions.pdf]

BMJ Open is committed to open peer review. As part of this commitment we make the peer review history of every article we publish publicly available.

When an article is published we post the peer reviewers' comments and the authors' responses online. We also post the versions of the paper that were used during peer review. These are the versions that the peer review comments apply to.

The versions of the paper that follow are the versions that were submitted during the peer review process. They are not the versions of record or the final published versions. They should not be cited or distributed as the published version of this manuscript.

BMJ Open is an open access journal and the full, final, typeset and author-corrected version of record of the manuscript is available on our site with no access controls, subscription charges or pay-per-view fees (http://bmjopen.bmj.com).

If you have any questions on BMJ Open's open peer review process please email info.bmjopen@bmj.com

BMJ Open

Development, deployment and evaluation of digitally-enabled, remote, supported rehabilitation for people with Long Covid (Living With Covid Recovery). Protocol for a mixed methods study.

Journal:	*BMJ Open*
Manuscript ID	bmjopen-2021-057408
Article Type:	Protocol
Date Submitted by the Author:	19-Sep-2021
Complete List of Authors:	Murray, Elizabeth; University College London, Research Department of Primary Care and Population Health<br>Goodfellow, Henry; University College London, Department of Primary Care and Population Health<br>Bindman, Julia; University College London, Research Department of Primary Care and Population Health<br>Blandford, Ann; University College London, UCLIC<br>Bradbury, Katherine; University of Southampton, Psychology<br>Chaudhry, Tahreem; University College London, Department of Primary Care and Population Health<br>Fernandez-Reyes, Delmiro; University College London, Computer Science<br>Gomes, Manuel; University College London, Department for Applied Health Research<br>Hamilton, Fiona; University College London, Research Department of Primary Care and Population Health<br>Heightman, Melissa; University College London Hospitals NHS Foundation Trust<br>Henley, W; University of Exeter Medical School, Institute of Health Sciences<br>Hurst, John; University College London, Academic Unit of Respiratory Medicine<br>Hylton, Hannah; Barts Health NHS Trust, Department of Respiratory medicine<br>Linke, Stuart; Camden and Islington NHS Foundation Trust<br>Pfeffer, Paul; Barts Health NHS Trust, Department of Respiratory Medicine<br>Ricketts, William; Barts Health NHS Trust, Respiratory Medicine<br>Robson, Chris; Living With<br>Singh, Richa; Barts Health NHS Trust, Department of Respiratory Medicine<br>Stevenson, Fiona; University College London, Research Department of Primary Care and Population Health<br>Walker, Sarah; University of Exeter<br>Waywell, Jonathan; Living With
Keywords:	COVID-19, REHABILITATION MEDICINE, Telemedicine < BIOTECHNOLOGY & BIOINFORMATICS, Health policy < HEALTH SERVICES ADMINISTRATION & MANAGEMENT

Title: Development, deployment and evaluation of digitally-enabled, remote, supported rehabilitation for people with Long Covid (Living With Covid Recovery). Protocol for a mixed methods study.

Authors:

Elizabeth Murray

Henry Goodfellow (henry.goodfellow@nhs.net)

Julia Bindman (juliabindman@jbsp.co.uk)

Ann Blandford (a.blandford@ucl.ac.uk)

Katherine Bradbury (k.bradbury@soton.ac.uk)

Tahreem Chaudhry (t.chaudhry@ucl.ac.uk)

Delmiro Fernandez-Reyes (delmiro.fernandez-reyes@ucl.ac.uk)

Manuel Gomes (m.gomes@ucl.ac.uk)

Fiona L. Hamilton (f.hamilton@ucl.ac.uk)

Melissa Heightman (melissa.heightman1@nhs.net)

William Henley (w.e.henley@exeter.ac.uk)

John R Hurst (j.hurst@ucl.ac.uk)

Hannah Hylton (h.hylton@nhs.net)

Stuart Linke (stuart.linke@candi.nhs.uk)

Paul Pfeffer (paul.pfeffer1@nhs.net)

Will Ricketts (william.ricketts@nhs.net)

Chris Robson (chris.robson@livingwith.health)

Richa Singh (richa.singh@nhs.net)

Fiona Stevenson (f.stevenson@ucl.ac.uk)

Sarah Walker (s.walker@exeter.ac.uk)

Jonathan Waywell (jonathan.waywell@livingwith.health).

Corresponding author:

Elizabeth Murray, FRCGP PhD. Elizabeth.murray@ucl.ac.uk

eHealth Unit, Research Department of Primary Care and Population Health, University College London

For peer review only

Abstract (285 words)

Introduction.

Long Covid is a distressing, disabling and heterogenous syndrome often causing severe functional impairment. Predominant symptoms include fatigue, cognitive impairment ("brain fog"), breathlessness and anxiety or depression. These symptoms are amenable to rehabilitation delivered by skilled healthcare professional), but Covid-19 has put severe strain on healthcare systems. This study aims to explore whether digitally-enabled, remotely supported rehabilitation for people with Long Covid can enable healthcare systems to provide high quality care to large numbers of patients within the available resources. Specific objectives are to a) develop and refine a digital health intervention (DHI) that supports patient assessment, monitoring and remote rehabilitation; b) develop implementation models that support sustainable deployment at scale; c) evaluate the impact of the DHI on recovery trajectories; and d) identify and mitigate health inequalities due to the digital divide.

Methods and Analysis.

Mixed methods, theoretically-informed, single arm prospective study, combining methods drawn from engineering / computer with those from biomedicine). There are four work packages (WP), one for each objective. WP1 focuses on identifying user requirements and iteratively developing the intervention to meet them; WP2 combines qualitative data from users with learning from implementation science and Normalisation Process Theory to promote adoption, scale-up, spread and sustainability of the intervention; WP3 uses quantitative demographic, clinical and resource use data collected by the DHI to determine illness trajectories and how these are affected by use of the DHI; while WP4 overarches the other three WP.

Ethics and Dissemination.

Ethical approval obtained from East Midlands – Derby Research Ethics Committee (reference 288199). Our dissemination strategy targets three audiences: (i) Policy makers, Health service managers and clinicians responsible for delivering Long Covid services; (ii) Patients and the public; (iii) Academics.

Registration.

Research Registry number: researchregistry6173

For peer review only

Article Summary

Strengths and limitations of this study.

- Strengths include our inter-disciplinary approach, which by combining methods common to engineering / computer science, e.g. User Centred Design and the Human Computer Interaction Lifecycle, with those from biomedicine, such as the MRC Complex Interventions Framework, promotes the likelihood of an intervention which is not only usable and acceptable, but also effective.
- Strong PPI input has helped define user requirements, including conceptualisation of Long Covid and focusing on function, fatigue and brain fog as well as ensuring the evaluation measures outcomes of interest to patients and clinicians.
- Intertwining service and research has enabled the rapid development and deployment of an evidence-based intervention, enabling treatment to reach patients rapidly during the pandemic.
- Our major limitation is the absence of a comparator group for the evaluation of effectiveness; our focus will be on profiling heterogeneity in recovery trajectories and conducting an exploratory mediation analysis to assess which intervention components and measures of app usage/engagement are associated with change in outcomes. In addition the results of this study inform the design and analysis of the STIMULATE-ICP project, which includes a cluster randomised controlled trial comparing LWCR to standard care.

For peer review only

For peer review only - http://bmjopen.bmj.com/site/about/guidelines.xhtml

Background

Since the emergence of the new coronavirus, SARS-CoV-2 (Severe Acute Respiratory Syndrome – Coronavirus -2) in Wuhan, China in December 2019 (1), it has become apparent that while many affected people make a full recovery, others are left with long-term disabling and distressing symptoms (2), known as “Long Covid” (3, 4). Prevalence estimates vary, but at least 50% of hospitalised (5, 6) and 10% of non-hospitalised continue to have symptoms 12 weeks after the initial infection (2, 4). UK Government figures suggest that by the end of July 2021, 5.9 million people in the UK had tested positive for Covid-19, of whom just under half a million (492,933) had been admitted to hospital (https://coronavirus.data.gov.uk/), suggesting that over 750,000 people in the UK may experience disabling symptoms persisting more than 12 weeks.

The patho-physiology of Long Covid has yet to be elucidated. It appears to be a multi-system disorder, characterised by variability in symptoms and a fluctuating trajectory (4), which significantly impairs people’s ability to work, look after their children, or engage with other activities. Despite variability in the nature and severity of reported symptoms, there are some core symptoms experienced by nearly all those with Long Covid: fatigue; cognitive impairment (“brain fog”); breathlessness; anxiety and depression (2). These core symptoms are present in numerous other long-term conditions, and treated with well established, non-pharmacological interventions, including physiotherapy, nutritional advice, cognitive behavioural approaches, sleep hygiene and improving self-management skills. These interventions are most often delivered by Allied Health Professionals (AHP), such as physiotherapists, occupational therapists, clinical psychologists, and dieticians, specialising in rehabilitation.

Prior to the Covid-19 pandemic, rehabilitation services were at capacity, and their ability to absorb over half a million additional patients requiring help has been further compromised by redeployment of many AHP to supporting acute services. Additionally, many services are system-focussed, so a patient with Long Covid who is breathless, fatigued and anxious could be directed to three different clinicians: a respiratory physiotherapist to help with breathing, a neuro-physiotherapist or occupational therapist for fatigue, and a clinical psychologist for anxiety, making treatment extremely burdensome (7).

One effect of the pandemic has been to rapidly accelerate the move toward the use of digital technologies to support health care (8). Digitally-supported rehabilitation, shown to be effective in other disease areas (9-12), has the potential to address the challenge of providing timely healthcare to large numbers of individuals with a highly constrained workforce (13). Digital health is not without its challenges: despite being shown to be effective in improving health outcomes, relatively few digital health interventions (DHI) have made it into mainstream healthcare (14, 15). There are well recognised problems with implementation at scale; many DHI suffer from poor uptake or inadequate ongoing use (16), reducing the likelihood of effectiveness. Furthermore, there are concerns around health inequalities and the digital divide, especially as Covid-19 disproportionately affects people from ethnic minority groups, socially deprived backgrounds and older people (17). People from these backgrounds are also less likely to have access to digital technology (18), so care delivered digitally risks exacerbating health inequalities.

Aims and objectives. The aim is to iteratively design, deploy and evaluate a digitally-mediated, remote, supported rehabilitation programme for patients affected by Covid-19, which is used effectively, deployed at scale, and does not widen health inequalities.

Specific objectives are to:

For peer review only - http://bmjopen.bmj.com/site/about/guidelines.xhtml

1. ***Develop and refine a DHI that supports patient monitoring, remote rehabilitation and identifying patients needing further specialist investigation***. To include:
   a) Clinical pathways aligned with NICE guidelines (19);
   b) A clinician-facing digital dashboard which displays data about individuals and selected cohorts;
   c) A patient-facing mobile app to provide targeted, tailored rehabilitation according to patient symptoms. The app will collect patient-reported outcome measures (PROMS) and use intelligent algorithms and machine learning to promote engagement and tailor treatment advice under clinician guidance.

2. ***Determine implementation strategies to promote adoption, scale-up, spread and sustainability with a view to maximising population impact.*** To include assessing:
   a) How best to integrate the DHI into clinical workflows, so it becomes fully normalised for healthcare professionals (HCP), and patients experience seamless care;
   b) The optimal amount of HCP input (time, skill set) required to support patients to engage effectively with the DHI, including those with low digital literacy, while managing large patient workloads safely;
   c) Funding and commissioning models to promote long-term sustainability.

3. ***Assess the population impact of this model of care.*** To include:
   a) Determining the reach (uptake and engagement) of the DHI, as a proportion of eligible patients and how this varies by demographic characteristics;
   b) Exploring its impact on patient-reported outcomes and recovery trajectories;
   c) Assessing the cost implications to the NHS of scaling-up and sustaining it.

4. ***Determine and mitigate the effect of the digital divide on health inequalities.***
   a) Identify patterns of differential uptake, use and apparent benefits of the DHI for people from ethnic minority or socially disadvantaged backgrounds;
   b) Identify and test actions to mitigate any observed differential.

Methods.

Overall Design: This project will combine research methods common to engineering and computer science (focused on developing a product that is safe, stable and meets user requirements) with those familiar to biomedical, behavioural and health service researchers (focused on effectiveness and population impact). Thus it will follow the Medical Research Council (MRC) Framework for development and evaluation of complex interventions (Phases 1, 2 and 4) (20), User-Centred Design (UCD), and the ISO 9241 Human-Computer Interaction (HCI) Lifecycle (21) for intervention development. Evaluation will use mixed methods, combining qualitative and quantitative data. The work will be divided into 4 work-packages (WP), with each WP addressing an objective (Figure 1).

Key principles: All work undertaken will adhere to two key principles:
   a) It addresses concurrently the aims of service and research, with the intention that the two should be mutually beneficial. The service priorities are to deliver a safe, effective and cost-effective service at speed in a resource-constrained environment; research requires us to generate new, generalisable knowledge.
   b) Inter-disciplinary approach with equal representation. The four communities represented in the team include: (i) patients, represented through Patient and Public Involvement (PPI); (ii) clinicians caring for patients with Long Covid, including Respiratory Specialists, General Practitioners, and Allied Health Professionals; (iii) industry, in the form of a small-medium enterprise (SME) specialising in digital health with substantial experience of deployment in

For peer review only - http://bmjopen.bmj.com/site/about/guidelines.xhtml

the NHS and other healthcare systems; (iv) academics. The academic disciplines represented in the team include Computer Science, Human-Computer Interaction, Digital Health, Behavioural Science, Social Science and implementation research, Statistics, Health economics, Health services research and Clinical academics.

Ethics. This protocol was approved by the East Midlands – Derby Research Ethics Committee (reference 288199).

Patient and Public Involvement (PPI). There is strong PPI representation throughout this study. The original application was developed with members of Long Covid social media groups, and the funding application was reviewed by PPI prior to submission. A PPI member (JB) is a co-investigator, named on the funding application, sits on the project steering committee and is a co-author on this paper. Once funding was confirmed, we recruited additional PPI members resulting in 2 PPI on the steering committee, 2 PPI on each of the work package groups, and a PPI Advisory Group. PPI members have contributed to: the overall design of the protocol; conceptualisation of Long Covid, including prioritisation of the symptoms of fatigue and brain fog; writing and reviewing the content, navigation and functionality of the intervention; selecting outcome measures; data collection, particularly running focus groups with other PPI; data analysis, particularly around user requirements; and continue to contribute to the study conduct.

Work Package 1. Development and refinement of the Living With Covid Recovery programme (LWCR).

Design: Inter-disciplinary team applying agile UCD and HCI methods to iteratively determine user requirements, to develop and refine the DHI by testing it against user requirements including optimising effectiveness in achieving desired behavioural changes (Figure 2).

Inputs include: user requirements; emerging clinical evidence on Long Covid; evidence-based treatments; development of personas (an HCI technique involving developing rich, contextualised descriptions of a range of potential users (14)); qualitative user feedback; and quantitative usage data. These data sources will be combined in an interative fashion, with 6 development cycles planned for the first year (Figure 2), starting with an existing platform designed for use in other health conditions.

Participants: We have identified three main stakeholder groups for user requirements: patients with Long Covid; HCP caring for patients with Long Covid; and health service managers responsible for Long Covid services. Initally, patient user requirements have been determined from PPI; recent ethics approval will allow data collection from patients using the LWCR programme. HCP and health service managers wil be identified initially through snowballing from the research team and subsequently from NHS teams using the service.

Data collection: We will use qualitative methods in real-time, including online meetings, interviews and focus groups with the three stakeholder groups, recorded with participant consent. Meetings will be driven by both research and service need to provide real-time insight into the needs of patients, health service managers and HCP, as they adapt to the evolving pandemic, without imposing additional workload. Documents, including meeting agendas and minutes, and follow-up emails, will also be used as data sources.

Quantitative data: see WP3. For ethical reasons we are unable to contact or collect data directly from patients who have declined the LWCR service.

For peer review only - http://bmjopen.bmj.com/site/about/guidelines.xhtml

Data analysis: Recordings will be stored securely. Relevant sections will be transcribed by a General Data Protection Regulation (GDPR) compliant transcribing company, anonymised, and checked for accuracy. Analysis will be done in two stages: first, a rapid, emergent analysis in parallel with data collection, focusing on actionable findings, fed immediately into the development process. Different data sources will be synthesised by the inter-disciplinary development team, through iterative discussions. Subsequent analysis will be in depth and inductive with transcripts coded by experienced qualitative researchers using NVivo, with additional deductive analysis where appropriate, driven by one of our theoretical frameworks or findings from the literature, critiqued in a multi-disciplinary data clinic.

Quantitative data will be anonymised. Initial analyses will be descriptive, with subsequent, more complex analyses focusing on usage patterns with a view to maximising uptake and use.

Outputs:

Evidence-based DHI which is rapidly and fully integrated into routine care pathways of NHS Long Covid clinics nationally. The DHI will be routinely updated every few months to increase usabiliy, engagement and effectiveness based on participant feedback.

Work Package 2: Determine implementation strategies to promote adoption, scale-up, spread and sustainability with a view to maximising population impact.

Design

Mixed methods, using quantitative data to measure uptake and use at individual and clinic level, and qualitative data to explore and understand barriers and facilitators to implementation. Data collection, analysis and development of implementation strategies will be informed by implementation science (15) and Normalisation Process Theory (NPT) (22).

Setting

We conceptualise implementation as occuring at the macro, meso and micro level. We define macro as system level change, such as change across multiple local health economies at national or regional level, for example NHS England (NHSE), Academic Health Science Networks (AHSNs) (23) or Integrated Care Systems (ICS) (24). Meso refers to change across a single clinic or local health economy; and micro refers to change at the level of the individual clinician or patient.

Participants

Participants for this WP include anyone with responsibility for implementing change in the delivery of care for patients with Long Covid at any level. This includes staff working for NHSE or the Department of Health and Social Care (DHSC) with responsibility for policy and practice; commissioners of care; local health service leaders, such as leads or staff working for AHSNs or ICS, clinical and managerial leads working within or across health service Trusts (acute, primary or community care), clinicians responsible for specific clinics and / or delivering front line care to patients with Long Covid, and patients with Long Covid or their carers. Participants also include professionals with particular relevant expertise needed to support the implementation of LWCR, such as understanding of Information Governance or procurement regulations in the NHS, and members of the research team with responsibility for setting up and delivering the service.

For peer review only - http://bmjopen.bmj.com/site/about/guidelines.xhtml

Data collection

Data collection and analysis will be shared across WP. Additional quantitative data collected for WP2 includes numbers of Trusts showing initial interest in deployment who decide against adoption, and demographic data on HCP registered on the dashboard, including clinical specialty, level of experience, age, gender and ethnicity, collected through a proforma questionnaire completed by each clinic once only.

Additional qualitative data will include email trails and documents published by DHSC, NHSE, AHSNs, ICS or other health service bodies. Clinician feedback on the service will be collected as part of the cycles of iterative development and refinement. This service-related data may be augmented, where necessary, by specific interviews with selected invidivuals, for example to obtain deeper insights into an issue raised in a meeting, or to test emerging interpretations of data. Where possible meetings will be recorded, with participant consent. Additional field notes and minutes will form part of the dataset.

Data pertaining to individual uptake and use of the LWCR programme will include feedback from clinicians, particularly focusing on how they introduce the service to patients and steps taken to encourage uptake and use, particularly amongst patients who are less used to digital technology. Opt-in consent will be sought from patients and clinicians to record specific on-boarding consultations. Patients will be invited to participate in interviews with the research team to explore their experience of Long Covid, the extent to which the service meets their perceived needs, and what could be done to improve the service for others.

Data analysis

Analysis of quantitative data is described in WP3. Qualitative data will be analysed in two stages, as described for WP1, with the first stage focusing on actionable findings to promote deployment and the second focusing on transferable learning about implementation of digital health interventions. In this second stage, data will be analysed inductively, with data-driven codes subsequently mapped onto NPT concepts, looking specifically for disconfirming data or data that does not map onto NPT.

Outputs from WP2 will include:

i) a fully developed implementation package, detailing the process of successful implementation of a digital health intervention into routine health care in a range of healthcare setting (primary, secondary and community care);
ii) a sustainable, scalable business model, allowing for ongoing maintenance and development of the product while it is used at scale;
iii) transferable learning about optimising the integration of digital health into routine care, ensuring optimal use by healthcare professionals and patients, including an understanding of how implementation models can mitigate the digital divide.

Work Package 3: Assess the population impact of this model of care.

Design: Quantitative workpackage. Population impact is defined as reach multiplied by effect.

Participants: Patients registered with LWCR.

For peer review only - http://bmjopen.bmj.com/site/about/guidelines.xhtml

Data collection: Quantitative data on numbers of patients, healthcare professionals and Long Covid clinics registered to use LWCR. Additionally, patient demographic data clinical and service data and app usage patterns, as recorded through the LWCR platform will be provided.

Demographic data will be obtained on patients' age, gender, ethnicity, highest level of educational attainment and socio-economic status (using Index of Multiple Deprivation, calculated from the postcode) when patients first register on the app. This data will be entered by healthcare professionals or the patients themselves.

Clinical data will be obtained from patient-completed Patient Reported Outcome Measures (PROMS) on the app. Patients will be asked to complete all the PROMS at baseline to help with clinical assessment, and thereafter, only those PROMS which the clinician and patient deem to be clinically relevant and helpful for clinical management.

Validated PROMS include: the Work and Social Adjustment Scale (WSAS) which measures functional impairment (25); EQ-5D-5L for health related quality of life (26); FACIT-F for fatigue (27); GAD-7 for anxiety (28); PHQ-8 for depression (29); MRC dyspnoea scale (21) and Dyspnoea-12 (30) for breathlessness; and PDQ-5 for cognitive impairment (31). Additional questions explore levels of physical activity and overall recovery from Covid-19.

Service use data will be obtained when patients complete a service use questionnaire (every 4 weeks), detailing the number of healthcare appointments, days off work and days as a hospital inpatient they have experienced.

Usage data, including the date and time of each page view by each patient are recorded automatically by the programme.

Data analysis

Each patient will be assigned a unique participant identification number (PIN). The linking key will be held by Living With, as data processors for the Clinical Trusts. Pseudonymised data, where date of birth is replaced by age and postcode replaced with IMD, will be shared with the research team. Demographic characteristics and baseline symptoms of the registered 'treatment seeking' population of long COVID sufferers will be profiled using descriptive statistics.

Usage and engagement. Patient engagement with the app will be reported. Factors influencing recruitment rates, uptake and use will be identified through logistic regression, with multivariate methods used to model patterns of app engagement over time.

Recovery trajectories. Generalised linear mixed models (GLMM) and latent class trajectory modelling (LCTM) will be used to examine stability and change in recovery trajectories for the longitudinal PROMS data, and to identify homogeneous clusters of patients with contrasting clincal phenotypes. Time since registration with the programme will initially be included as a linear covariate, but we will explore the functional form of the effect of time to allow for phenomena such as a plateau in symptom recovery or a period of sudden rapid improvement or relapse. Models will include random slopes and intercepts, allowing for individualised differences in severity of baseline symptoms and subsequent trajectories. Fixed effects will include age, gender, ethnicity, IMD and educational attainment.

Mediation analysis of intervention components. A mediation analysis will be undertaken in which intervention components and measures of patient app usage and engagement will be added to

For peer review only - http://bmjopen.bmj.com/site/about/guidelines.xhtml

longitudinal models as explanatory factors. We will assess whether there is a dose-response relationship between usage of the app / introduction of new content and change in outcomes.

Effectiveness of the LWCR programme. We will investigate the feasibility of creating a synthetic comparator arm from patients with long Covid enrolled in suitable comparator cohorts, hosted in BREATHE-SAIL (32). Direct comparisons will be made between patients receiving the evidence-based interventions and the comparator group(s) in multivariable GLMMs with propensity score methods used to provide control for measured confounders.

Equality. The acceptability of the intervention for people from disadvantaged backgrounds will be explored through logistic regression analysis of these factors as predictors of recruitment, usage and retention. Differential effects of the DHI on outcomes for disadvantaged groups will be investigated through inclusion of interactions between intervention uptake and ethnicity, age, educational status, and IMD in the GLMMs.

Missing data. Sensitivity analyses will be conducted to examine the influence of missing data on the key study findings. This will include the use of multiple imputation methods, where the assumption that the data are missing at random (MAR) is considered appropriate, and tipping point analysis (33) to assess sensitivity of the results to data that are missing not at random (MNAR).

Health economic evaluation.

The health economic evaluation will estimate the cost of delivering the DHI to the target population, the cost consequences of the DHI for the NHS services compared to standard care, and a budget impact analysis over the next 3-5 years (34) that assesses the costs and potential savings resulting from commissioning the proposed DHI. To generate costs from the resource use data, appropriate unit costs; e.g. price lists from the app developer to cost items related to app development, and Unit Costs for Health and Social Care (35) to cost staff time, will be applied.

Relevant costs will be identified by examining how the DHI impacts the patient, hospital, and the health care system. These will include:

- Resources associated with the development and hosting of the patient-facing app.
- Resources incurred with the scaling up the DHI, such as maintenance, updating, data storage, implementation, and training.
- Resource use associated with HCP support of patients using the service.
- Impact on health service use.

Outputs.

Outputs from WP3 will include detailed understanding of patient symptoms and illness trajectories over time, estimates of the impact of the digital health programme on these trajectories and an understanding of the relative cost-effectiveness of the LWCR programme compared with standard care.

Work package 4: Determine and mitigate the effect of the digital divide on health inequalities.

Design. This cross-cutting work package will work across the other three, ensuring that the work undertaken is fully sensitive to the needs of minority ethnic groups, socially disadvantaged or older people, identifies evidence of the digital divide and health inequalities, and acts to mitigate these. Examples of input into work package 1 include ensuring that the personas used in development

For peer review only - http://bmjopen.bmj.com/site/about/guidelines.xhtml

include minority ethnic, socially disadvantaged and older people, that the content is written for a reading age of entry level 3 (attained by over 80% of the UK population) (36) and a systematic scoping review to identify features of DHI design and deployment that enable engagement by people with low digital/health literacy, e.g. key content is presented using graphics, video, and audio to enhance accessibility. Input into WP2 will include identifying and exploring factors in the implementation processes (including input by health care professionals and use of digital champions or other support teams to facilitate widening access). In WP3, input will include identifying the influence of demographic factors on differential uptake, use and impact.

Outputs. An intervention which is accessible to people from a range of demographic backgrounds, an understanding of the features of the implementation model which promote participation by disadvantaged people, and data on any differential uptake or impact of the digital health intervention according to demographic characteristics.

Discussion.

This protocol describes the processes for developing, deploying and evaluating digitally-enabled remote, supported rehabilitation for people with Long Covid. Strengths include the inter-disciplinary approach, with a partnership between patients, clinicians, industry and academics, plus combining approaches used in engineering / computer science, e.g. UCD and the HCI lifecycle, with those more familiar to biomedical researchers, e.g. the MRC Framework for complex interventions. The strong use of theory to underpin the research should enhance transferability of findings. Putting equal priority on service needs and research is innovative, but brings challenges. These include relying on diversity of service delivery for obtaining an appropriate comparator and the reliance on data obtained for clinical purposes for the impact evaluation. We expect there to be substantial amounts of missing data, particularly in the PROMS, as clinicians and patients are likely to focus on recording symptoms of most interest to them. However, no one study can answer all relevant research questions about an innovation, and we agree with the argument that a digital health intervention should achieve a reasonable level of stability before being evaluated in a randomised controlled trial (37). This initial study will provide considerable insight into patient, clinician and healthservice requirements for digitally-supported rehabilitation for Long Covid, together with very substantial, real world data on acceptability, uptake and use, going far beyond the usual pilot studies so often used as evidence of acceptability and feasibility, with data on scalable, and hopefully, sustainable, deployment. It will also generate data on symptoms and illness trajectories in a treatment-seeking population which includes non-hospitalised patients, making it unique amongst current cohort studies. Long term investment will require causal evidence of effectiveness, and NIHR have funded the STIMULATE-ICP project, which includes a cluster randomised controlled trial comparing LWCR to standard care (38), due to report in 2023.

In conclusion, we suggest that our approach could help alleviate the well known delay in translating research findings into practice (39) whilst simultaneously promoting the sustainable, scalable adoption of evidence-based interventions and adding to the research base in digital health.

For peer review only - http://bmjopen.bmj.com/site/about/guidelines.xhtml

For peer review only

Author Contributions.

All authors, with the exception of TC and SW, contributed the development of the successful funding application on which this protocol is based. TC further developed and operationalised the protocol as part of the ethics submission and SW elaborated the statistical analyses. EM and HG are co-chief investigators. EM wrote the first and final draft of the paper. All authors have read and approved the final version.

Funding statement.

This report is independent research funded by the National Institute for Health Research (NIHR Health Services and Delivery Research, NIHR132243 - Supported remote rehabilitation post Covid-19: development, deployment and evaluation of a digitally-enabled rehabilitation programme). The views expressed in this publication are those of the author(s) and not necessarily those of the National Institute for Health Research or the Department of Health and Social Care.

Competing interests statement.

Chris Robson and Jonathan Waywell are directors of the provider company, *Living With*, which has a suite of digital health interventions used in healthcare systems including the NHS.

Data Statement

Data generated by this study will be deposited with the Breathe-Sail database.

Word Count: 3,953

References

1. Guan WJ, Ni ZY, Hu Y, Liang WH, Ou CQ, He JX, et al. Clinical Characteristics of Coronavirus Disease 2019 in China. The New England journal of medicine. 2020;382(18):1708-20.
2. Crook H, Raza S, Nowell J, Young M, Edison P. Long covid—mechanisms, risk factors, and management. BMJ (Clinical research ed). 2021;374:n1648.
3. Castaranes-Zapatero DH, G; van den Heede, K; . Epidemiology of long COVID: a pragmatic review of the literature. Brussels: KCE; 2021 27/01/21.
4. NIHR. Living With Covid19. A dynamic review of the evidence around ongoing Covid19 (often called Long Covid). London; 2021 16/03/2021.
5. Mandal S, Barnett J, Brill SE, Brown JS, Denneny EK, Hare SS, et al. 'Long-COVID': a cross-sectional study of persisting symptoms, biomarker and imaging abnormalities following hospitalisation for COVID-19. Thorax. 2021;76(4):396-8.
6. Taylor RR, Trivedi B, Patel N, Singh R, Ricketts WM, Elliott K, et al. Post-COVID symptoms reported at asynchronous virtual review and stratified follow-up after COVID-19 pneumonia. Clinical medicine (London, England). 2021;21(4):e384-91.
7. May CR, Eton DT, Boehmer K, Gallacher K, Hunt K, MacDonald S, et al. Rethinking the patient: using Burden of Treatment Theory to understand the changing dynamics of illness. BMC health services research. 2014;14:281.
8. Hutchings R. The impact of Covid-19 on the use of digital technology in the NHS. Briefing August 2020. London; 2020.

For peer review only - http://bmjopen.bmj.com/site/about/guidelines.xhtml

9. Dunphy E, Button K, Hamilton F, Williams J, Spasic I, Murray E. Feasibility randomised controlled trial comparing TRAK-ACL digital rehabilitation intervention plus treatment as usual versus treatment as usual for patients following anterior cruciate ligament reconstruction. BMJ open sport & exercise medicine. 2021;7(2):e001002.
10. Geraghty AWA, Stanford R, Stuart B, Little P, Roberts LC, Foster NE, et al. Using an internet intervention to support self-management of low back pain in primary care: findings from a randomised controlled feasibility trial (SupportBack). BMJ open. 2018;8(3):e016768.
11. Bourne S, DeVos R, North M, Chauhan A, Green B, Brown T, et al. Online versus face-to-face pulmonary rehabilitation for patients with chronic obstructive pulmonary disease: randomised controlled trial. BMJ open. 2017;7(7):e014580.
12. Widmer RJ, Allison TG, Lennon R, Lopez-Jimenez F, Lerman LO, Lerman A. Digital health intervention during cardiac rehabilitation: A randomized controlled trial. Am Heart J. 2017;188:65-72.
13. Providers N. SPOTLIGHT ON... DIGITAL TRANSFORMATION IN RESPONSE TO COVID-19. 2020.
14. Greenhalgh T, Wherton J, Papoutsi C, Lynch J, Hughes G, A'Court C, et al. Beyond Adoption: A New Framework for Theorizing and Evaluating Nonadoption, Abandonment, and Challenges to the Scale-Up, Spread, and Sustainability of Health and Care Technologies. Journal of medical Internet research. 2017;19(11):e367.
15. Ross J, Stevenson F, Lau R, Murray E. Factors that influence the implementation of e-Helath: A systematic review of systematic reviews (an update). Implementation science : IS. 2016.
16. Perski O, Blandford A, West R, Michie S. Conceptualising engagement with digital behaviour change interventions: a systematic review using principles from critical interpretive synthesis. Translational behavioral medicine. 2017;7(2):254-67.
17. Patel JA, Nielsen FBH, Badiani AA, Assi S, Unadkat VA, Patel B, et al. Poverty, inequality and COVID-19: the forgotten vulnerable. Public Health 2020;183:110-1.
18. Powell CB, L. Digital Divide: Mapping the geodemographics of internet accessibility across Great Britain: arXiv; 2021 [Available from: https://arxiv.org/abs/2108.07699.
19. NICE. Covid-19 Rapid Guideline: Managing Covid-19. London; 2021 10/08/21.
20. Craig P, Dieppe P, Macintyre S, Michie S, Nazareth I, Petticrew M. Developing and evaluating complex interventions: the new Medical Research Council guidance. BMJ. 2008;337:a1655. doi: 10.1136/bmj.a1655.:a1655.
21. Sharp H, Preece J, Rogers Y. Interaction Design: Beyond Human-Computer Interaction. 5th ed: Wiley; 2019.
22. May C, Finch T. Implementation, embedding, and integration: an outline of Normalization Process Theory. Sociology. 2009;43(3):535-54.
23. Network TA. Academic Health Science Networks: Transforming lives through healthcare innovation 2021 [Available from: https://www.ahsnnetwork.com/.
24. England N. What is Integrated Care? 2021 [Available from: https://www.england.nhs.uk/integratedcare/what-is-integrated-care/.
25. Mundt JC, Marks IM, Shear MK, Greist JH. The Work and Social Adjustment Scale: a simple measure of impairment in functioning. The British journal of psychiatry : the journal of mental science. 2002;180:461-4.
26. Herdman M, Gudex C, Lloyd A, Janssen M, Kind P, Parkin D, et al. Development and preliminary testing of the new five-level version of EQ-5D (EQ-5D-5L). Quality of life research : an international journal of quality of life aspects of treatment, care and rehabilitation. 2011;20(10):1727-36.
27. Lai JS, Cella D, Choi S, Junghaenel DU, Christodoulou C, Gershon R, et al. How item banks and their application can influence measurement practice in rehabilitation medicine: a PROMIS fatigue item bank example. Archives of physical medicine and rehabilitation. 2011;92(10 Suppl):S20-7.
28. Spitzer RL, Kroenke K, Williams JB, Löwe B. A brief measure for assessing generalized anxiety disorder: the GAD-7. Arch Intern Med. 2006;166.

For peer review only - http://bmjopen.bmj.com/site/about/guidelines.xhtml

29. Kroenke K, Strine TW, Spitzer RL, Williams JBW, Berry JT, Mokdad AH. The PHQ-8 as a measure of current depression in the general population. J Affective Disorders. 2009;114.
30. Yorke J, Swigris J, Russell AM, Moosavi SH, Ng Man Kwong G, Longshaw M, et al. Dyspnea-12 is a valid and reliable measure of breathlessness in patients with interstitial lung disease. Chest. 2011;139(1):159-64.
31. Julian LJ, Yazdany J, Trupin L, Criswell LA, Yelin E, Katz PP. Validity of brief screening tools for cognitive impairment in rheumatoid arthritis and systemic lupus erythematosus. Arthritis care & research. 2012;64(3):448-54.
32. UK HDR. Breathe: Our Hub for Respiratory Health 2021 [Available from: https://www.hdruk.ac.uk/helping-with-health-data/our-hubs-across-the-uk/breathe/.
33. Yan X, Lee S, Li N. Missing data handling methods in medical device clinical trials. Journal of biopharmaceutical statistics. 2009;19(6):1085-98.
34. Assessing resource impact manual. London; 2021.
35. Curtis LB, A. . Unit Costs of Health and Social Care 2020. Canterbury: Personal Social Services Research Unit, University of Kent; 2020.
36. Trust L. What do adult literacy levels mean? 2021 [Available from: https://literacytrust.org.uk/parents-and-families/adult-literacy/what-do-adult-literacy-levels-mean/), .
37. Murray E, Hekler EB, Andersson G, Collins LM, Doherty A, Hollis C, et al. Evaluating Digital Health Interventions: Key Questions and Approaches. American journal of preventive medicine. 2016;51(5):843-51.
38. New research into treatment and diagnosis of long COVID [press release]. 18.07.2021 2021.
39. Grol R, Grimshaw J. From best evidence to best practice: effective implementation of change in patients' care. Lancet. 2003;362(9391):1225-30.

For peer review only

For peer review only - http://bmjopen.bmj.com/site/about/guidelines.xhtml

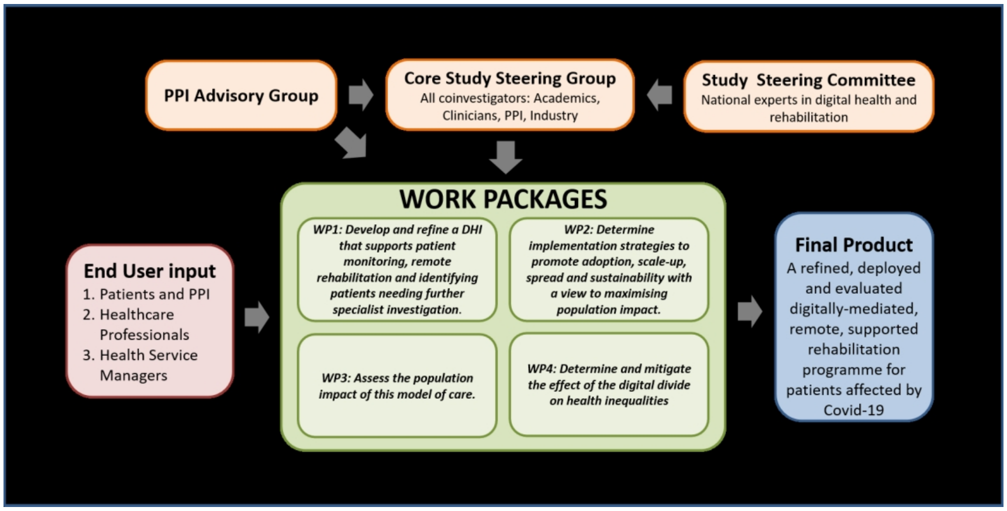

Figure 1 Living With Covid Recovery Organisational Structure

146x73mm (300 x 300 DPI)

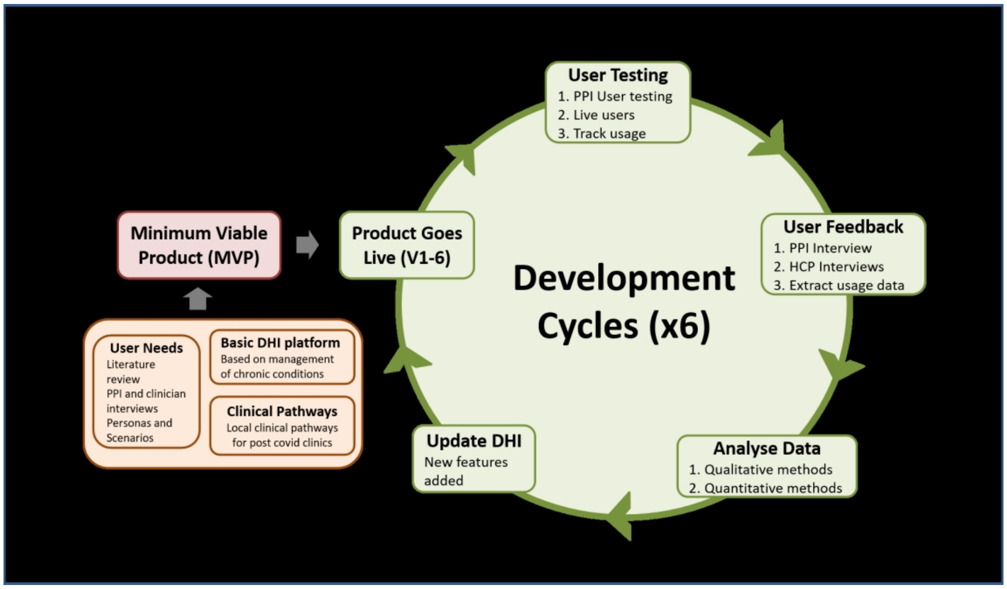

Figure 2 Iterative cycles of development and refinement for Living With Covid Recovery

159x92mm (300 x 300 DPI)

BMJ Open

Development, deployment and evaluation of digitally-enabled, remote, supported rehabilitation for people with Long Covid (Living With Covid Recovery). Protocol for a mixed methods study.

Journal:	*BMJ Open*
Manuscript ID	bmjopen-2021-057408.R1
Article Type:	Protocol
Date Submitted by the Author:	15-Nov-2021

Complete List of Authors:	Murray, Elizabeth; University College London, Research Department of Primary Care and Population Health<br>Goodfellow, Henry; University College London, Department of Primary Care and Population Health<br>Bindman, Julia; University College London, Research Department of Primary Care and Population Health<br>Blandford, Ann; University College London, UCLIC<br>Bradbury, Katherine; University of Southampton, Psychology<br>Chaudhry, Tahreem; University College London, Department of Primary Care and Population Health<br>Fernandez-Reyes, Delmiro; University College London, Computer Science<br>Gomes, Manuel; University College London, Department for Applied Health Research<br>Hamilton, Fiona; University College London, Research Department of Primary Care and Population Health<br>Heightman, Melissa; University College London Hospitals NHS Foundation Trust<br>Henley, W; University of Exeter Medical School, Institute of Health Sciences<br>Hurst, John; University College London, Academic Unit of Respiratory Medicine<br>Hylton, Hannah; Barts Health NHS Trust, Department of Respiratory medicine<br>Linke, Stuart; Camden and Islington NHS Foundation Trust<br>Pfeffer, Paul; Barts Health NHS Trust, Department of Respiratory Medicine<br>Ricketts, William; Barts Health NHS Trust, Respiratory Medicine<br>Robson, Chris; Living With<br>Singh, Richa; Barts Health NHS Trust, Department of Respiratory Medicine<br>Stevenson, Fiona; University College London, Research Department of Primary Care and Population Health<br>Walker, Sarah; University of Exeter<br>Waywell, Jonathan; Living With

**Primary Subject Heading**:	Rehabilitation medicine

Secondary Subject Heading:	Research methods
Keywords:	COVID-19, REHABILITATION MEDICINE, Telemedicine < BIOTECHNOLOGY & BIOINFORMATICS, Health policy < HEALTH SERVICES ADMINISTRATION & MANAGEMENT

SCHOLARONE™
Manuscripts

Title: Development, deployment and evaluation of digitally-enabled, remote, supported rehabilitation for people with Long Covid (Living With Covid Recovery). Protocol for a mixed methods study.

Authors:

Elizabeth Murray

Henry Goodfellow (henry.goodfellow@nhs.net)

Julia Bindman (juliabindman@jbsp.co.uk)

Ann Blandford (a.blandford@ucl.ac.uk)

Katherine Bradbury (k.bradbury@soton.ac.uk)

Tahreem Chaudhry (t.chaudhry@ucl.ac.uk)

Delmiro Fernandez-Reyes (delmiro.fernandez-reyes@ucl.ac.uk)

Manuel Gomes (m.gomes@ucl.ac.uk)

Fiona L. Hamilton (f.hamilton@ucl.ac.uk)

Melissa Heightman (melissa.heightman1@nhs.net)

William Henley (w.e.henley@exeter.ac.uk)

John R Hurst (j.hurst@ucl.ac.uk)

Hannah Hylton (h.hylton@nhs.net)

Stuart Linke (stuart.linke@candi.nhs.uk)

Paul Pfeffer (paul.pfeffer1@nhs.net)

Will Ricketts (william.ricketts@nhs.net)

Chris Robson (chris.robson@livingwith.health)

Richa Singh (richa.singh@nhs.net)

Fiona Stevenson (f.stevenson@ucl.ac.uk)

Sarah Walker (s.walker@exeter.ac.uk)

Jonathan Waywell (jonathan.waywell@livingwith.health).

Corresponding author:

Elizabeth Murray, FRCGP PhD. Elizabeth.murray@ucl.ac.uk

eHealth Unit, Research Department of Primary Care and Population Health, University College London

For peer review only - http://bmjopen.bmj.com/site/about/guidelines.xhtml

Abstract (294 words)

Introduction.

Long Covid is a distressing, disabling and heterogenous syndrome often causing severe functional impairment. Predominant symptoms include fatigue, cognitive impairment ("brain fog"), breathlessness and anxiety or depression. These symptoms are amenable to rehabilitation delivered by skilled healthcare professional, but Covid-19 has put severe strain on healthcare systems. This study aims to explore whether digitally-enabled, remotely supported rehabilitation for people with Long Covid can enable healthcare systems to provide high quality care to large numbers of patients within the available resources. Specific objectives are to a) develop and refine a digital health intervention (DHI) that supports patient assessment, monitoring and remote rehabilitation; b) develop implementation models that support sustainable deployment at scale; c) evaluate the impact of the DHI on recovery trajectories; and d) identify and mitigate health inequalities due to the digital divide.

Methods and Analysis.

Mixed methods, theoretically-informed, single arm prospective study, combining methods drawn from engineering / computer science with those from biomedicine. There are four Work Packages (WP), one for each objective. WP1 focuses on identifying user requirements and iteratively developing the intervention to meet them; WP2 combines qualitative data from users with learning from implementation science and Normalisation Process Theory to promote adoption, scale-up, spread and sustainability of the intervention; WP3 uses quantitative demographic, clinical and resource use data collected by the DHI to determine illness trajectories and how these are affected by use of the DHI; while WP4 focuses on identifying and mitigating health inequalities and overarches the other three WPs.

Ethics and Dissemination.

Ethical approval obtained from East Midlands – Derby Research Ethics Committee (reference 288199). Our dissemination strategy targets three audiences: (i) Policy makers, Health service managers and clinicians responsible for delivering Long Covid services; (ii) Patients and the public; (iii) Academics.

Registration.

Research Registry number: researchregistry6173

For peer review only - http://bmjopen.bmj.com/site/about/guidelines.xhtml

Article Summary

Strengths and limitations of this study.

- Strengths include our inter-disciplinary approach, which by combining methods common to engineering / computer science, e.g. User Centred Design and the Human Computer Interaction Lifecycle, with those from biomedicine, such as the MRC Complex Interventions Framework, promotes the likelihood of an intervention which is not only usable and acceptable, but also effective.
- Strong Patient and Public Involvement (PPI) input has helped define user requirements, including conceptualisation of Long Covid and focusing on function, fatigue and brain fog as well as ensuring the evaluation measures outcomes of interest to patients and clinicians.
- Intertwining service and research has enabled the rapid development and deployment of an evidence-based intervention, enabling treatment to reach patients rapidly during the pandemic.
- Our major limitation is the absence of a comparator group for the evaluation of effectiveness; our focus will be on profiling heterogeneity in recovery trajectories and conducting an exploratory mediation analysis to assess which intervention components and measures of app usage/engagement are associated with change in outcomes. In addition, the results of this study inform the design and analysis of the STIMULATE-ICP project, which includes a cluster randomised controlled trial comparing LWCR to standard care.

For peer review only - http://bmjopen.bmj.com/site/about/guidelines.xhtml

Background

Since the emergence of the new coronavirus, SARS-CoV-2 (Severe Acute Respiratory Syndrome – Coronavirus -2) in Wuhan, China in December 2019 (1), it has become apparent that while many affected people make a full recovery, others are left with long-term disabling and distressing symptoms (2), known as "Long Covid" (3, 4). Prevalence estimates vary, but at least 50% of hospitalised (5, 6) and 10% of non-hospitalised continue to have symptoms 12 weeks after the initial infection (2, 4). UK Government figures suggest that by the end of July 2021, 5.9 million people in the UK had tested positive for Covid-19, of whom just under half a million (492,933) had been admitted to hospital (https://coronavirus.data.gov.uk/), suggesting that over 750,000 people in the UK may experience disabling symptoms persisting more than 12 weeks.

The patho-physiology of Long Covid has yet to be elucidated. It appears to be a multi-system disorder, characterised by variability in symptoms and a fluctuating trajectory (4), which significantly impairs people's ability to work, look after their children, or engage with other activities. Despite variability in the nature and severity of reported symptoms, there are some core symptoms experienced by nearly all those with Long Covid: fatigue; cognitive impairment ("brain fog"); breathlessness; anxiety and depression (2). These core symptoms are present in numerous other long-term conditions, and treated with well established, non-pharmacological interventions, including physiotherapy, nutritional advice, cognitive behavioural approaches, sleep hygiene and improving self-management skills. These interventions are most often delivered by Allied Health Professionals (AHP), such as physiotherapists, occupational therapists, clinical psychologists, and dieticians, specialising in rehabilitation.

Prior to the Covid-19 pandemic, rehabilitation services were at capacity, and their ability to absorb over half a million additional patients requiring help has been further compromised by redeployment of many AHP to supporting acute services. Additionally, many services are system-focussed, so a patient with Long Covid who is breathless, fatigued and anxious could be directed to three different clinicians: a respiratory physiotherapist to help with breathing, a neuro-physiotherapist or occupational therapist for fatigue, and a clinical psychologist for anxiety, making treatment extremely burdensome (7).

One effect of the pandemic has been to rapidly accelerate the move toward the use of digital technologies to support health care (8). Digitally-supported rehabilitation, shown to be effective in other disease areas (9-12), has the potential to address the challenge of providing timely healthcare to large numbers of individuals with a highly constrained workforce (13). Digital health is not without its challenges: despite being shown to be effective in improving health outcomes, relatively few digital health interventions (DHI) have made it into mainstream healthcare (14, 15). There are well recognised problems with implementation at scale; many DHI suffer from poor uptake or inadequate ongoing use (16), reducing the likelihood of effectiveness. Furthermore, there are concerns around health inequalities and the digital divide, especially as Covid-19 disproportionately affects people from ethnic minority groups, socially deprived backgrounds and older people (17). People from these backgrounds are also less likely to have access to digital technology (18), so care delivered digitally risks exacerbating health inequalities. However, it has been shown that the digital divide can be overcome by careful design and implementation into routine health care (19).

Aims and objectives. The aim is to iteratively design, deploy and evaluate a digitally-mediated, remote, supported rehabilitation programme for patients affected by Covid-19, which is used effectively, deployed at scale, and does not widen health inequalities.

For peer review only - http://bmjopen.bmj.com/site/about/guidelines.xhtml

Specific objectives are to:

1. ***Develop and refine a DHI that supports patient monitoring, remote rehabilitation and identifying patients needing further specialist investigation***. To include:
    a) Clinical pathways aligned with NICE guidelines (20);
    b) A clinician-facing digital dashboard which displays data about individuals and selected cohorts;
    c) A patient-facing mobile app to provide targeted, tailored rehabilitation according to patient symptoms. The app will collect patient-reported outcome measures (PROMS) and use intelligent algorithms and machine learning to promote engagement and tailor treatment advice under clinician guidance.

2. ***Determine implementation strategies to promote adoption, scale-up, spread and sustainability with a view to maximising population impact.*** To include assessing:
    a) How best to integrate the DHI into clinical workflows, so it becomes fully normalised for healthcare professionals (HCP), and patients experience seamless care;
    b) The optimal amount of HCP input (time, skill set) required to support patients to engage effectively with the DHI, including those with low digital literacy, while managing large patient workloads safely;
    c) Funding and commissioning models to promote long-term sustainability.

3. ***Assess the population impact of this model of care.*** To include:
    a) Determining the reach (uptake and engagement) of the DHI, as a proportion of eligible patients and how this varies by demographic characteristics;
    b) Exploring its impact on patient-reported outcomes and recovery trajectories;
    c) Assessing the cost implications to the NHS of scaling-up and sustaining it.

4. ***Determine and mitigate the effect of the digital divide on health inequalities.***
    a) Identify patterns of differential uptake, use and apparent benefits of the DHI for people from ethnic minority or socially disadvantaged backgrounds;
    b) Identify and test actions to mitigate any observed differential.

Methods.

Overall Design: This project will combine research methods common to engineering and computer science (focused on developing a product that is safe, stable and meets user requirements) with those familiar to biomedical, behavioural and health service researchers (focused on effectiveness and population impact). Thus it will follow the Medical Research Council (MRC) Framework for development and evaluation of complex interventions (Phases 1, 2 and 4) (21), User-Centred Design (UCD), and the ISO 9241 Human-Computer Interaction (HCI) Lifecycle (22) for intervention development. Evaluation will use mixed methods, combining qualitative and quantitative data. The work will be divided into 4 work-packages (WP) across 2 years, with each WP addressing an objective (Figure 1).

Key principles: All work undertaken will adhere to two key principles:

a) It addresses concurrently the aims of service and research, with the intention that the two should be mutually beneficial. The service priorities are to deliver a safe, effective and cost-effective service at speed in a resource-constrained environment; research requires us to generate new, generalisable knowledge.

For peer review only - http://bmjopen.bmj.com/site/about/guidelines.xhtml

b) Inter-disciplinary approach with equal representation. The four communities represented in the team include: (i) patients, represented through Patient and Public Involvement (PPI); (ii) clinicians caring for patients with Long Covid, including Respiratory Specialists, General Practitioners, and Allied Health Professionals; (iii) industry, in the form of a small-medium enterprise (SME) specialising in digital health with substantial experience of deployment in the NHS and other healthcare systems; (iv) academics. The academic disciplines represented in the team include Computer Science, Human-Computer Interaction, Digital Health, Behavioural Science, Social Science and implementation research, Statistics, Health economics, Health services research and Clinical academics.

Patient and Public Involvement (PPI). There is strong PPI representation throughout this study. The original application was developed with members of Long Covid social media groups, and the funding application was reviewed by PPI prior to submission. A PPI member (JB) is a co-investigator, named on the funding application, sits on the project steering committee and is a co-author on this paper. Once funding was confirmed, we recruited additional PPI members resulting in 2 PPI on the steering committee, 2 PPI on each of the work package groups, and a PPI Advisory Group. PPI members have contributed to: the overall design of the protocol; conceptualisation of Long Covid, including prioritisation of the symptoms of fatigue and brain fog; writing and reviewing the content, navigation and functionality of the intervention; selecting outcome measures; data collection, particularly running focus groups with other PPI; data analysis, particularly around user requirements; and continue to contribute to the study conduct.

Timeline. This is a two year project with WP1 mainly focused on the first year of the project to design and optimise the product. WP3 will mainly be focused on the second year. WP2 & 4 will run throughout both years.

Work Package 1. Development and refinement of the Living With Covid Recovery programme (LWCR).

Design: Inter-disciplinary team applying agile User Centred Design (UCD) and Human Computer Interaction (HCI) methods to iteratively determine user requirements, to develop and refine the DHI by testing it against user requirements including optimising effectiveness in achieving desired behavioural changes (Figure 2).

Inputs include: user requirements; emerging clinical evidence on Long Covid; evidence-based treatments; development of personas (an HCI technique involving developing rich, contextualised descriptions of a range of potential users (14)); qualitative user feedback; and quantitative usage data. These data sources will be combined in an interative fashion, with 6 development cycles planned for the first year (Figure 2), starting with an existing platform designed for use in other chronic health conditions such as cancer, rhumatoid arthritis and incontinence.

Participants: We have identified three main stakeholder groups for user requirements: patients with Long Covid; Health Care Professionals (HCP) caring for patients with Long Covid; and health service managers responsible for Long Covid services. Initally, patient user requirements have been determined from PPI; recent ethics approval will allow data collection from patients using the LWCR programme. HCP and health service managers wil be identified initially through snowballing from the research team and subsequently from NHS teams using the service.

Data collection: We will use qualitative methods in real-time, including online meetings, interviews and focus groups with the three stakeholder groups, recorded with participant consent. Meetings

For peer review only - http://bmjopen.bmj.com/site/about/guidelines.xhtml

will be driven by both research and service need to provide real-time insight into the needs of patients, health service managers and HCP, as they adapt to the evolving pandemic, without imposing additional workload. Documents, including meeting agendas and minutes, and follow-up emails, will also be used as data sources.

Quantitative data: see WP3. For ethical reasons we are unable to contact or collect data directly from patients who have declined the LWCR service.

Data analysis: Recordings will be stored securely. Relevant sections will be transcribed by a General Data Protection Regulation (GDPR) compliant transcribing company, anonymised, and checked for accuracy. Analysis will be done in two stages: first, a rapid, emergent analysis in parallel with data collection, focusing on actionable findings, fed immediately into the development process. Different data sources will be synthesised by the inter-disciplinary development team, through iterative discussions. Subsequent analysis will be in depth and inductive with transcripts coded by experienced qualitative researchers using NVivo, with additional deductive analysis where appropriate, driven by one of our theoretical frameworks or findings from the literature, critiqued in a multi-disciplinary data clinic.

Quantitative data will be anonymised. Initial analyses will be descriptive, with subsequent, more complex analyses focusing on usage patterns with a view to maximising uptake and use.

Outputs:

Evidence-based DHI which is rapidly and fully integrated into routine care pathways of NHS Long Covid clinics nationally. The DHI will be routinely updated with new features for the App and Dashboard such as questionnaries or content every few months to increase usabiliy, engagement and effectiveness based on participant feedback. Patients and Clinicians will be made aware of changes through notifications on the DHI or emails and supported with a helpline.

Work Package 2: Determine implementation strategies to promote adoption, scale-up, spread and sustainability with a view to maximising population impact.

Design

Mixed methods, using quantitative data to measure uptake and use at individual and clinic level, and qualitative data to explore and understand barriers and facilitators to implementation. Data collection, analysis and development of implementation strategies will be informed by Normalisation Process Theory (NPT) (15) (23), a sociological theory concerned with the work required to implement, embed and integrate (or "normalise") new practices or technologies into routine healthcare.

Setting

The LWCR programme will be implemented in community Long Covid clinics nationally; these clinics will cover both urban and rural areas. The use of the LWCR programme is free for any NHS long covid clinic in England until September 2022. We conceptualise implementation as occuring at the macro, meso and micro level. We define macro as system level change, such as change across multiple local health economies at national or regional level, for example NHS England (NHSE), Academic Health Science Networks (AHSNs) (24) or Integrated Care Systems (ICS) (25). Meso refers to change across a single clinic or local health economy; and micro refers to change at the level of the individual clinician or patient.

For peer review only - http://bmjopen.bmj.com/site/about/guidelines.xhtml

Participants

Participants for this WP include anyone with responsibility for implementing change in the delivery of care for patients with Long Covid at any level. This includes staff working for NHSE or the Department of Health and Social Care (DHSC) with responsibility for policy and practice; commissioners of care; local health service leaders, such as leads or staff working for AHSNs or ICS, clinical and managerial leads working within or across health service Trusts (acute, primary or community care), clinicians responsible for specific clinics and / or delivering front line care to patients with Long Covid, and patients with Long Covid or their carers. Participants also include professionals with particular relevant expertise needed to support the implementation of LWCR, such as understanding of Information Governance or procurement regulations in the NHS, and members of the research team with responsibility for setting up and delivering the service.

Data collection

Data collection and analysis will be shared across WPs. Additional quantitative data collected for WP2 includes numbers of Trusts showing initial interest in deployment who decide against adoption, and demographic data on HCP registered on the dashboard, including clinical specialty, level of experience, age, gender and ethnicity, collected through a proforma questionnaire completed by each clinic once only.

Additional qualitative data will include email trails and documents published by DHSC, NHSE, AHSNs, ICS or other health service bodies. Clinician feedback on the service will be collected as part of the cycles of iterative development and refinement. This service-related data may be augmented, where necessary, by specific interviews with selected invidivuals, for example to obtain deeper insights into an issue raised in a meeting, or to test emerging interpretations of data. Where possible meetings will be recorded, with participant consent. Field notes and minutes will also form part of the dataset.

Data pertaining to individual uptake and use of the LWCR programme will include feedback from clinicians, particularly focusing on how they introduce the service to patients and steps taken to encourage uptake and use, particularly amongst patients who are less used to digital technology. Opt-in consent will be sought from patients and clinicians to record specific on-boarding consultations. Patients will be invited to participate in interviews with the research team to explore their experience of Long Covid, the extent to which the service meets their perceived needs, and what could be done to improve the service for others.

Data analysis

Analysis of quantitative data is described in WP3. Qualitative data will be analysed in two stages, as described for WP1, with the first stage focusing on actionable findings to promote deployment and the second focusing on transferable learning about implementation of digital health interventions. In this second stage, data will be analysed inductively, with data-driven codes subsequently mapped onto NPT concepts, looking specifically for disconfirming data or data that does not map onto NPT.

Outputs from WP2 will include:

For peer review only - http://bmjopen.bmj.com/site/about/guidelines.xhtml

i) a fully developed implementation package, detailing the process of successful implementation of a digital health intervention into routine health care in a range of healthcare setting (primary, secondary and community care);
ii) a sustainable, scalable business model, allowing for ongoing maintenance and development of the product while it is used at scale;
iii) transferable learning about optimising the integration of digital health into routine care, ensuring optimal use by healthcare professionals and patients, including an understanding of how implementation models can mitigate the digital divide.

Work Package 3: Assess the population impact of this model of care.

Design: Quantitative workpackage. Population impact is defined as reach multiplied by effect.

Participants: The target for year 1 will be to recruit 1,000 patients registering with the DHI. This will enable us to estimate the overall proportion of patients engaging with the app with a high-level of precision (maximum width of 95% confidence interval is +/- 3.2%). Similar or increased levels of precision will be achieved when estimating the proportion of those referred who register with the intervention. The sample size in year 2 has not been pre-specified and will depend upon practical constraints imposed by the scale of the pandemic as the digital intervention is rolled out nationally. Power calculations will be carried out for the year 2 analyses based on the 'pilot' data from year 1. This will include power for the proposed quasi-experimental analysis of the effect of the DHI on outcomes at 3- and 6-months follow-up.

Data collection:
Quantitative data on numbers of patients, healthcare professionals and Long Covid clinics registered to use LWCR. Additionally, patient demographic data clinical and service data and app usage patterns, as recorded through the LWCR platform will be provided.

Demographic data will be obtained on patients' age, gender, ethnicity, highest level of educational attainment and socio-economic status (using Index of Multiple Deprivation, calculated from the postcode) when patients first register on the app. This data will be entered by healthcare professionals or the patients themselves.

Clinical data will be obtained from patient-completed Patient Reported Outcome Measures (PROMS) on the app. Patients will be asked to complete all the PROMS at baseline to help with clinical assessment, and thereafter, only those PROMS which the clinician and patient deem to be clinically relevant and helpful for clinical management.

Validated PROMS include: the Work and Social Adjustment Scale (WSAS) which measures functional impairment and is our primary outcome measure (26); EQ-5D-5L for health related quality of life (27); FACIT-F for fatigue (28); GAD-7 for anxiety (29); PHQ-8 for depression (30); MRC dyspnoea scale (22) and Dyspnoea-12 (31) for breathlessness; and PDQ-5 for cognitive impairment (32). Additional questions explore levels of physical activity and overall recovery from Covid-19.

Service use data will be obtained when patients complete a service use questionnaire (every 4 weeks), detailing the number of healthcare appointments, days off work and days as a hospital inpatient they have experienced.

Usage data, including the date and time of each page view by each patient are recorded automatically by the programme.

For peer review only - http://bmjopen.bmj.com/site/about/guidelines.xhtml

Data analysis

Each patient will be assigned a unique participant identification number (PIN). The linking key will be held by Living With, as data processors for the Clinical Trusts. Pseudonymised data, where date of birth is replaced by age and postcode replaced with IMD, will be shared with the research team. Demographic characteristics and baseline symptoms of the registered 'treatment seeking' population of long COVID sufferers will be profiled using descriptive statistics.

Usage and engagement. Patient engagement with the app will be reported using a stratification of patients into low, medium and high categories based on numbher of log ins over the first 12 weeks. Demographic and baseline clinical factors influencing uptake and use will be identified through logistic regression, with multivariate methods used to model patterns of app engagement over time.

Recovery trajectories. Generalised linear mixed models (GLMM) and latent class trajectory modelling (LCTM) will be used to examine stability and change in recovery trajectories for the longitudinal PROMS data, and to identify homogeneous clusters of patients with contrasting clincal phenotypes. Time since registration with the programme will initially be included as a linear covariate, but we will explore the functional form of the effect of time to allow for phenomena such as a plateau in symptom recovery or a period of sudden rapid improvement or relapse. Models will include random slopes and intercepts, allowing for individualised differences in severity of baseline symptoms and subsequent trajectories. Fixed effects will include age, gender, ethnicity, IMD and educational attainment.

Mediation analysis of intervention components. A mediation analysis will be undertaken in which intervention components and measures of patient app usage and engagement (number of log ins and assessment completed) will be added to longitudinal models as explanatory factors. We will explore whether increased engagment by clinicians such as messages sent and reviews of patient assessments on the dashboard is linked with improved patient outcomes. We will assess whether there is a dose-response relationship between usage of the app / introduction of new content and change in outcomes.

Effectiveness of the LWCR programme. We will investigate the feasibility of creating a synthetic comparator arm from patients with long Covid enrolled in suitable comparator cohorts, hosted in BREATHE-SAIL (33). Direct comparisons will be made between patients receiving the evidence-based interventions and the comparator group(s) in multivariable GLMMs with propensity score methods used to provide control for measured confounders.

Equality. The acceptability of the intervention for people from disadvantaged backgrounds will be explored through logistic regression analysis of these factors as predictors of recruitment, usage and retention. Differential effects of the DHI on outcomes for disadvantaged groups will be investigated through inclusion of interactions between intervention uptake and ethnicity, age, educational status, and IMD in the GLMMs.

Missing data. Sensitivity analyses will be conducted to examine the influence of missing data on the key study findings. This will include the use of multiple imputation methods, where the assumption that the data are missing at random (MAR) is considered appropriate, and tipping point analysis (34) to assess sensitivity of the results to data that are missing not at random (MNAR).

Health economic evaluation.

For peer review only - http://bmjopen.bmj.com/site/about/guidelines.xhtml

The health economic evaluation will estimate the cost of delivering the DHI to the target population, the cost consequences of the DHI for the NHS services compared to standard care, and a budget impact analysis over the next 3-5 years (35) that assesses the costs and potential savings resulting from commissioning the proposed DHI. To generate costs from the resource use data, appropriate unit costs; e.g. price lists from the app developer to cost items related to app development, and Unit Costs for Health and Social Care (36) to cost staff time, will be applied.

Relevant costs will be identified by examining how the DHI impacts the patient, hospital, and the health care system. These will include:

- Resources associated with the development and hosting of the patient-facing app.
- Resources incurred with the scaling up the DHI, such as maintenance, updating, data storage, implementation, and training.
- Resource use associated with HCP support of patients using the service.
- Impact on health service use.

Outputs.

Outputs from WP3 will include detailed understanding of patient symptoms and illness trajectories over time, estimates of the impact of the digital health programme on these trajectories and an understanding of the relative cost-effectiveness of the LWCR programme compared with standard care i.e. the care provided to those patients not supported by the LWCR programme.

Work Package 4: Determine and mitigate the effect of the digital divide on health inequalities.

Design. This cross-cutting WP will ensure that the work undertaken in the other three WPs is fully sensitive to the needs of those who might be at risk of exclusion, such as minority ethnic groups, socially disadvantaged or older people. It will seek to identify evidence of the digital divide and health inequalities, and work to mitigate these. We will undertake a systematic scoping review to identify features of DHI design and deployment that enable engagement by people with low digital/health literacy, e.g. key content is presented using graphics, video, and audio to enhance accessibility.

In relation to WP1 the focus will be on ensuring that the personas used in developmental design include minority ethnic, socially disadvantaged, and older people, to improve usability and engagement in these groups. We will ensure the content is written for a reading age of entry level 3 (attained by over 80% of the UK population) (37) and therefore understandable by most patients. Design features will allow family or carers to log in and support patients. The systematic scoping review will inform intervention design and deployment to maximise engagement by people with low digital/health literacy.

In relation to WP2 the focus will be on identifying and exploring factors in the implementation process that could hinder uptake and increase health inequalities, or conversely, help to mitigate inequalities. For example, translation of key content into different languages, the use of digital champions to help support patients, referral to charities that supply smart phones and advise how to access low-cost data or free Wi-Fi to help facilitate increased access to all groups.

In relation to WP3 the focus will be on ensuring the analysis of the outcome measures can be compared across different demographic groups so that disparities can be identified. Exploring the factors that can influence differential uptake, use, and impact across the different clinics and demographic groups.

For peer review only - http://bmjopen.bmj.com/site/about/guidelines.xhtml

Outputs. An intervention which is accessible to people from a range of demographic backgrounds and that collects/analyses/acts on data suggesting differential uptake or impact of the digital health intervention by people from these demographic groups; and an understanding of the features of the implementation model which promote participation by people from ethnic minority, disadvantaged, or older groups.

Ethics and dissemination

This protocol was approved by the East Midlands – Derby Research Ethics Committee (reference 288199). Ethical considerations was given to obtaining patient consent and maintaining patient anonimity. First consent to contact patients by email was obtained using a tick box on the App. Only those patietns that opted in will be contacted and ask if they wish to participate in interviews. All patient data collected from the LWCR product was psudeo-anonymised by Livign With. Patient email addresses from those who agreed to be contacted will be stored separately in the universtiy data safe haven in accordance with the GDPR and data goverance protocols.

Our dissemination strategy targets three audiences: (i) Policy makers, Health service managers and clinicians responsible for delivering Long Covid services; (ii) Patients and the public; (iii) Academics. Findings will be disseminated at conferences and papers to target academics, through nationally advertised workshops for clinicians and administrators working in or running long covid clinics, and through news and social media for patients.

Discussion.

This protocol describes the processes for developing, deploying and evaluating digitally-enabled remote, supported rehabilitation for people with Long Covid. Strengths include the inter-disciplinary approach, with a partnership between patients, clinicians, industry and academics, plus combining approaches used in engineering / computer science, e.g. UCD and the HCI lifecycle, with those more familiar to biomedical researchers, e.g. the MRC Framework for complex interventions. The strong use of theory to underpin the research should enhance transferability of findings. Putting equal priority on service needs and research is innovative, but brings challenges. These include relying on diversity of service delivery for obtaining an appropriate comparator and the reliance on data obtained for clinical purposes for the impact evaluation. We expect there to be substantial amounts of missing data, particularly in the PROMS, as clinicians and patients are likely to focus on recording symptoms of most interest to them. However, no one study can answer all relevant research questions about an innovation, and we agree with the argument that a digital health intervention should achieve a reasonable level of stability before being evaluated in a randomised controlled trial (38). This initial study will provide considerable insight into patient, clinician and healthservice requirements for digitally-supported rehabilitation for Long Covid, together with very substantial, real world data on acceptability, uptake and use, going far beyond the usual pilot studies so often used as evidence of acceptability and feasibility, with data on scalable, and hopefully, sustainable, deployment. It will also generate data on symptoms and illness trajectories in a treatment-seeking population which includes non-hospitalised patients, making it unique amongst current cohort studies. Long term investment will require causal evidence of effectiveness, and NIHR have funded the STIMULATE-ICP project, which includes a cluster randomised controlled trial comparing LWCR to standard care (39), due to report in 2023.

For peer review only - http://bmjopen.bmj.com/site/about/guidelines.xhtml

In conclusion, we suggest that our approach could help alleviate the well known delay in translating research findings into practice (40) whilst simultaneously promoting the sustainable, scalable adoption of evidence-based interventions and adding to the research base in digital health.

For peer review only

For peer review only - http://bmjopen.bmj.com/site/about/guidelines.xhtml

For peer review only

Author Contributions.

All authors (EM, HG, JB, AB, KB, DFR, MG, FH, MH, WH, JH, HH, SL, PP, WR, CR, RS, FS and JW), with the exception of TC and SW, contributed to the development of the successful funding application on which this protocol is based. TC further developed and operationalised the protocol as part of the ethics submission and SW elaborated the statistical analyses. EM and HG are co-chief investigators and JB, AB, KB, DFR, MG, FH, MH, WH, JH, HH, SL, PP, WR, CR, RS, FS and JW are co-investigators on the project. EM wrote the first draft of the paper and incorporated comments on the draft manuscript from all authors. HG wrote the response to the reviewer comments after discussion and comments from all authors. All authors have read and approved the final version.

Funding statement.

This study is funded by the National Institute for Health Research (NIHR) Crossprogramme [HS&DR] COVID-19 [project reference NIHR132243 - Supported remote rehabilitation post Covid-19: development, deployment and evaluation of a digitally-enabled rehabilitation programme]. The views expressed are those of the author(s) and not necessarily those of the NIHR or the Department of Health and Social Care.
This report is independent research supported by the National Institute for Health Research ARC North Thames.

Competing interests statement.

Chris Robson and Jonathan Waywell are directors of the provider company, *Living With*, which has a suite of digital health interventions used in healthcare systems including the NHS.

Data Statement

Data generated by this study will be deposited with the Breathe-Sail database.

Word Count: 3,953

References

1. Guan WJ, Ni ZY, Hu Y, Liang WH, Ou CQ, He JX, et al. Clinical Characteristics of Coronavirus Disease 2019 in China. The New England journal of medicine. 2020;382(18):1708-20.
2. Crook H, Raza S, Nowell J, Young M, Edison P. Long covid—mechanisms, risk factors, and management. BMJ (Clinical research ed). 2021;374:n1648.
3. Castaranes-Zapatero DH, G; van den Heede, K; . Epidemiology of long COVID: a pragmatic review of the literature. Brussels: KCE; 2021 27/01/21.
4. NIHR. Living With Covid19. A dynamic review of the evidence around ongoing Covid19 (often called Long Covid). London; 2021 16/03/2021.
5. Mandal S, Barnett J, Brill SE, Brown JS, Denneny EK, Hare SS, et al. 'Long-COVID': a cross-sectional study of persisting symptoms, biomarker and imaging abnormalities following hospitalisation for COVID-19. Thorax. 2021;76(4):396-8.
6. Taylor RR, Trivedi B, Patel N, Singh R, Ricketts WM, Elliott K, et al. Post-COVID symptoms reported at asynchronous virtual review and stratified follow-up after COVID-19 pneumonia. Clinical medicine (London, England). 2021;21(4):e384-91.

For peer review only - http://bmjopen.bmj.com/site/about/guidelines.xhtml

7. May CR, Eton DT, Boehmer K, Gallacher K, Hunt K, MacDonald S, et al. Rethinking the patient: using Burden of Treatment Theory to understand the changing dynamics of illness. BMC health services research. 2014;14:281.
8. Hutchings R. The impact of Covid-19 on the use of digital technology in the NHS. Briefing August 2020. London; 2020.
9. Dunphy E, Button K, Hamilton F, Williams J, Spasic I, Murray E. Feasibility randomised controlled trial comparing TRAK-ACL digital rehabilitation intervention plus treatment as usual versus treatment as usual for patients following anterior cruciate ligament reconstruction. BMJ open sport & exercise medicine. 2021;7(2):e001002.
10. Geraghty AWA, Stanford R, Stuart B, Little P, Roberts LC, Foster NE, et al. Using an internet intervention to support self-management of low back pain in primary care: findings from a randomised controlled feasibility trial (SupportBack). BMJ open. 2018;8(3):e016768.
11. Bourne S, DeVos R, North M, Chauhan A, Green B, Brown T, et al. Online versus face-to-face pulmonary rehabilitation for patients with chronic obstructive pulmonary disease: randomised controlled trial. BMJ open. 2017;7(7):e014580.
12. Widmer RJ, Allison TG, Lennon R, Lopez-Jimenez F, Lerman LO, Lerman A. Digital health intervention during cardiac rehabilitation: A randomized controlled trial. Am Heart J. 2017;188:65-72.
13. Providers N. SPOTLIGHT ON... DIGITAL TRANSFORMATION IN RESPONSE TO COVID-19. 2020.
14. Greenhalgh T, Wherton J, Papoutsi C, Lynch J, Hughes G, A'Court C, et al. Beyond Adoption: A New Framework for Theorizing and Evaluating Nonadoption, Abandonment, and Challenges to the Scale-Up, Spread, and Sustainability of Health and Care Technologies. Journal of medical Internet research. 2017;19(11):e367.
15. Ross J, Stevenson F, Lau R, Murray E. Factors that influence the implementation of e-Helath: A systematic review of systematic reviews (an update). Implementation science : IS. 2016.
16. Perski O, Blandford A, West R, Michie S. Conceptualising engagement with digital behaviour change interventions: a systematic review using principles from critical interpretive synthesis. Translational behavioral medicine. 2017;7(2):254-67.
17. Patel JA, Nielsen FBH, Badiani AA, Assi S, Unadkat VA, Patel B, et al. Poverty, inequality and COVID-19: the forgotten vulnerable. Public Health 2020;183:110-1.
18. Powell CB, L. Digital Divide: Mapping the geodemographics of internet accessibility across Great Britain: arXiv; 2021 [Available from: https://arxiv.org/abs/2108.07699.
19. Poduval S, Ahmed S, Marston L, Hamilton F, Murray E. Crossing the Digital Divide in Online Self-Management Support: Analysis of Usage Data From HeLP-Diabetes. JMIR Diabetes. 2018;3(4):e10925.
20. NICE. Covid-19 Rapid Guideline: Managing Covid-19. London; 2021 10/08/21.
21. Craig P, Dieppe P, Macintyre S, Michie S, Nazareth I, Petticrew M. Developing and evaluating complex interventions: the new Medical Research Council guidance. BMJ. 2008;337:a1655. doi: 10.1136/bmj.a1655.:a1655.
22. Sharp H, Preece J, Rogers Y. Interaction Design: Beyond Human-Computer Interaction. 5th ed: Wiley; 2019.
23. May C, Finch T. Implementation, embedding, and integration: an outline of Normalization Process Theory. Sociology. 2009;43(3):535-54.
24. Network TA. Academic Health Science Networks: Transforming lives through healthcare innovation 2021 [Available from: https://www.ahsnnetwork.com/.
25. England N. What is Integrated Care? 2021 [Available from: https://www.england.nhs.uk/integratedcare/what-is-integrated-care/.
26. Mundt JC, Marks IM, Shear MK, Greist JH. The Work and Social Adjustment Scale: a simple measure of impairment in functioning. The British journal of psychiatry : the journal of mental science. 2002;180:461-4.

For peer review only

For peer review only - http://bmjopen.bmj.com/site/about/guidelines.xhtml

27. Herdman M, Gudex C, Lloyd A, Janssen M, Kind P, Parkin D, et al. Development and preliminary testing of the new five-level version of EQ-5D (EQ-5D-5L). Quality of life research : an international journal of quality of life aspects of treatment, care and rehabilitation. 2011;20(10):1727-36.
28. Lai JS, Cella D, Choi S, Junghaenel DU, Christodoulou C, Gershon R, et al. How item banks and their application can influence measurement practice in rehabilitation medicine: a PROMIS fatigue item bank example. Archives of physical medicine and rehabilitation. 2011;92(10 Suppl):S20-7.
29. Spitzer RL, Kroenke K, Williams JB, Löwe B. A brief measure for assessing generalized anxiety disorder: the GAD-7. Arch Intern Med. 2006;166.
30. Kroenke K, Strine TW, Spitzer RL, Williams JBW, Berry JT, Mokdad AH. The PHQ-8 as a measure of current depression in the general population. J Affective Disorders. 2009;114.
31. Yorke J, Swigris J, Russell AM, Moosavi SH, Ng Man Kwong G, Longshaw M, et al. Dyspnea-12 is a valid and reliable measure of breathlessness in patients with interstitial lung disease. Chest. 2011;139(1):159-64.
32. Julian LJ, Yazdany J, Trupin L, Criswell LA, Yelin E, Katz PP. Validity of brief screening tools for cognitive impairment in rheumatoid arthritis and systemic lupus erythematosus. Arthritis care & research. 2012;64(3):448-54.
33. UK HDR. Breathe: Our Hub for Respiratory Health 2021 [Available from: https://www.hdruk.ac.uk/helping-with-health-data/our-hubs-across-the-uk/breathe/.
34. Yan X, Lee S, Li N. Missing data handling methods in medical device clinical trials. Journal of biopharmaceutical statistics. 2009;19(6):1085-98.
35. Assessing resource impact manual. London; 2021.
36. Curtis LB, A. . Unit Costs of Health and Social Care 2020. Canterbury: Personal Social Services Research Unit, University of Kent; 2020.
37. Trust L. What do adult literacy levels mean? 2021 [Available from: https://literacytrust.org.uk/parents-and-families/adult-literacy/what-do-adult-literacy-levels-mean/), .
38. Murray E, Hekler EB, Andersson G, Collins LM, Doherty A, Hollis C, et al. Evaluating Digital Health Interventions: Key Questions and Approaches. American journal of preventive medicine. 2016;51(5):843-51.
39. New research into treatment and diagnosis of long COVID [press release]. 18.07.2021 2021.
40. Grol R, Grimshaw J. From best evidence to best practice: effective implementation of change in patients' care. Lancet. 2003;362(9391):1225-30.

For peer review only

For peer review only - http://bmjopen.bmj.com/site/about/guidelines.xhtml

Figure Legend:

Figure 1: Living With Covid Recovery Organisational Structure

Figure 2: Iterative cycles of development and refinement for Living With Covid Recovery

For peer review only

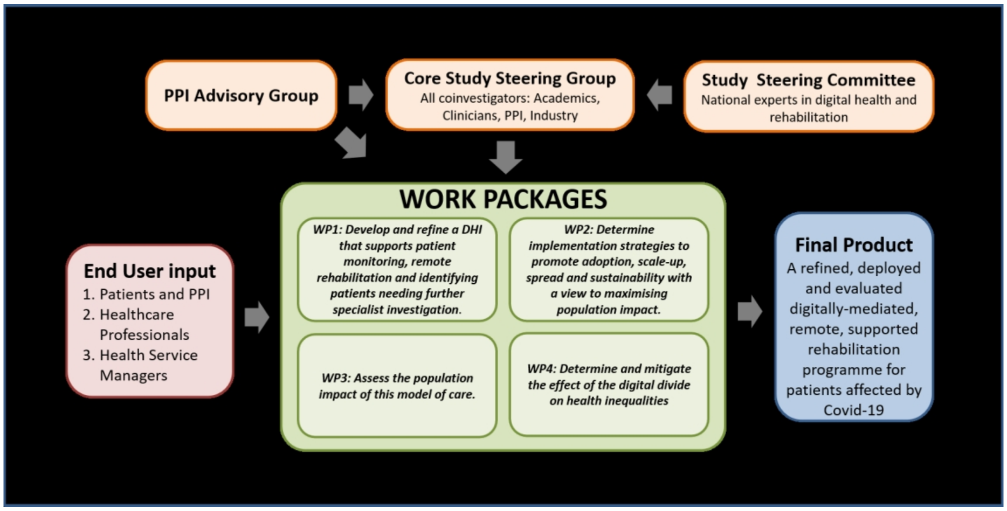

Figure 1 Living With Covid Recovery Organisational Structure

146x73mm (300 x 300 DPI)

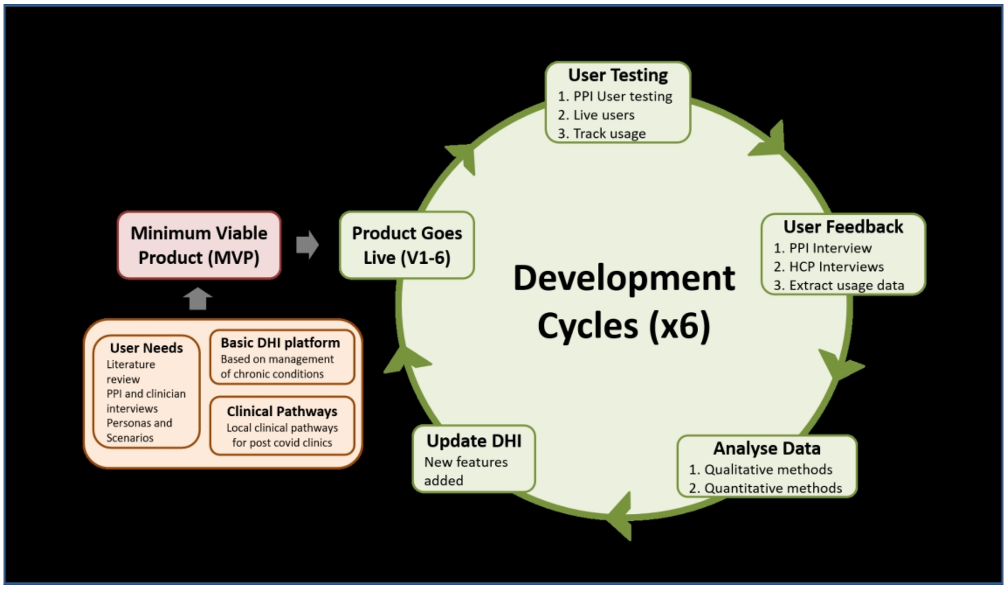

Figure 2 Iterative cycles of development and refinement for Living With Covid Recovery

159x92mm (300 x 300 DPI)

BMJ Open

Development, deployment and evaluation of digitally-enabled, remote, supported rehabilitation for people with Long Covid (Living With Covid Recovery). Protocol for a mixed methods study.

Journal:	*BMJ Open*
Manuscript ID	bmjopen-2021-057408.R2
Article Type:	Protocol
Date Submitted by the Author:	16-Dec-2021

Complete List of Authors:	Murray, Elizabeth; University College London, Research Department of Primary Care and Population Health<br>Goodfellow, Henry; University College London, Department of Primary Care and Population Health<br>Bindman, Julia; University College London, Research Department of Primary Care and Population Health<br>Blandford, Ann; University College London, UCLIC<br>Bradbury, Katherine; University of Southampton, Psychology<br>Chaudhry, Tahreem; University College London, Department of Primary Care and Population Health<br>Fernandez-Reyes, Delmiro; University College London, Computer Science<br>Gomes, Manuel; University College London, Department for Applied Health Research<br>Hamilton, Fiona; University College London, Research Department of Primary Care and Population Health<br>Heightman, Melissa; University College London Hospitals NHS Foundation Trust<br>Henley, W; University of Exeter Medical School, Institute of Health Sciences<br>Hurst, John; University College London, Academic Unit of Respiratory Medicine<br>Hylton, Hannah; Barts Health NHS Trust, Department of Respiratory medicine<br>Linke, Stuart; Camden and Islington NHS Foundation Trust<br>Pfeffer, Paul; Barts Health NHS Trust, Department of Respiratory Medicine<br>Ricketts, William; Barts Health NHS Trust, Respiratory Medicine<br>Robson, Chris; Living With<br>Singh, Richa; Barts Health NHS Trust, Department of Respiratory Medicine<br>Stevenson, Fiona; University College London, Research Department of Primary Care and Population Health<br>Walker, Sarah; University of Exeter<br>Waywell, Jonathan; Living With

**Primary Subject Heading**:	Rehabilitation medicine

Secondary Subject Heading:	Research methods
Keywords:	COVID-19, REHABILITATION MEDICINE, Telemedicine < BIOTECHNOLOGY & BIOINFORMATICS, Health policy < HEALTH SERVICES ADMINISTRATION & MANAGEMENT

SCHOLARONE™
Manuscripts

Title: Development, deployment and evaluation of digitally-enabled, remote, supported rehabilitation for people with Long Covid (Living With Covid Recovery). Protocol for a mixed methods study.

Authors:

Elizabeth Murray

Henry Goodfellow (henry.goodfellow@nhs.net)

Julia Bindman (juliabindman@jbsp.co.uk)

Ann Blandford (a.blandford@ucl.ac.uk)

Katherine Bradbury (k.bradbury@soton.ac.uk)

Tahreem Chaudhry (t.chaudhry@ucl.ac.uk)

Delmiro Fernandez-Reyes (delmiro.fernandez-reyes@ucl.ac.uk)

Manuel Gomes (m.gomes@ucl.ac.uk)

Fiona L. Hamilton (f.hamilton@ucl.ac.uk)

Melissa Heightman (melissa.heightman1@nhs.net)

William Henley (w.e.henley@exeter.ac.uk)

John R Hurst (j.hurst@ucl.ac.uk)

Hannah Hylton (h.hylton@nhs.net)

Stuart Linke (stuart.linke@candi.nhs.uk)

Paul Pfeffer (paul.pfeffer1@nhs.net)

Will Ricketts (william.ricketts@nhs.net)

Chris Robson (chris.robson@livingwith.health)

Richa Singh (richa.singh@nhs.net)

Fiona Stevenson (f.stevenson@ucl.ac.uk)

Sarah Walker (s.walker@exeter.ac.uk)

Jonathan Waywell (jonathan.waywell@livingwith.health).

Corresponding author:

Elizabeth Murray, FRCGP PhD. Elizabeth.murray@ucl.ac.uk

eHealth Unit, Research Department of Primary Care and Population Health, University College London

For peer review only

Abstract (294 words)

Introduction.

Long Covid is a distressing, disabling and heterogenous syndrome often causing severe functional impairment. Predominant symptoms include fatigue, cognitive impairment ("brain fog"), breathlessness and anxiety or depression. These symptoms are amenable to rehabilitation delivered by skilled healthcare professionals, but Covid-19 has put severe strain on healthcare systems. This study aims to explore whether digitally-enabled, remotely supported rehabilitation for people with Long Covid can enable healthcare systems to provide high quality care to large numbers of patients within the available resources. Specific objectives are to a) develop and refine a digital health intervention (DHI) that supports patient assessment, monitoring and remote rehabilitation; b) develop implementation models that support sustainable deployment at scale; c) evaluate the impact of the DHI on recovery trajectories; and d) identify and mitigate health inequalities due to the digital divide.

Methods and Analysis.

Mixed methods, theoretically-informed, single arm prospective study, combining methods drawn from engineering / computer science with those from biomedicine. There are four Work Packages (WP), one for each objective. WP1 focuses on identifying user requirements and iteratively developing the intervention to meet them; WP2 combines qualitative data from users with learning from implementation science and Normalisation Process Theory to promote adoption, scale-up, spread and sustainability of the intervention; WP3 uses quantitative demographic, clinical and resource use data collected by the DHI to determine illness trajectories and how these are affected by use of the DHI; while WP4 focuses on identifying and mitigating health inequalities and overarches the other three WPs.

Ethics and Dissemination.

Ethical approval obtained from East Midlands – Derby Research Ethics Committee (reference 288199). Our dissemination strategy targets three audiences: (i) Policy makers, Health service managers and clinicians responsible for delivering Long Covid services; (ii) Patients and the public; (iii) Academics.

Registration.

Research Registry number: researchregistry6173

For peer review only - http://bmjopen.bmj.com/site/about/guidelines.xhtml

Article Summary

Strengths and limitations of this study.

- Strengths include our inter-disciplinary approach, which by combining methods common to engineering / computer science, e.g. User Centred Design and the Human Computer Interaction Lifecycle, with those from biomedicine, such as the Medical Research Council (MRC) Complex Interventions Framework, promotes the likelihood of an intervention which is not only usable and acceptable, but also effective.
- Strong Patient and Public Involvement (PPI) input has helped define user requirements, including conceptualisation of Long Covid and focusing on function, fatigue and brain fog as well as ensuring the evaluation measures outcomes of interest to patients and clinicians.
- Intertwining service and research has enabled the rapid development and deployment of an evidence-based intervention, enabling treatment to reach patients rapidly during the pandemic.
- Our major limitation is the absence of a comparator group for the evaluation of effectiveness; our focus will be on profiling heterogeneity in recovery trajectories and conducting an exploratory mediation analysis to assess which intervention components and measures of app usage/engagement are associated with change in outcomes..

For peer review only

Background

Since the emergence of the new coronavirus, SARS-CoV-2 (Severe Acute Respiratory Syndrome – Coronavirus -2) in Wuhan, China in December 2019 (1) and the subsequent COVID-19 variants, it has become apparent that while many affected people make a full recovery, others are left with long-term disabling and distressing symptoms (2), known as “Long Covid syndrome” (3, 4). Prevalence estimates vary, but at least 50% of hospitalised (5, 6) and 10% of non-hospitalised continue to have symptoms 12 weeks after the initial infection (2, 4). UK Government figures suggest that by the end of July 2021, 5.9 million people in the UK had tested positive for Covid-19, of whom just under half a million (492,933) had been admitted to hospital (https://coronavirus.data.gov.uk/), suggesting that over 750,000 people in the UK may experience disabling symptoms persisting more than 12 weeks.

The patho-physiology of Long Covid has yet to be elucidated. It appears to be a multi-system disorder, characterised by variability in symptoms and a fluctuating trajectory (4), which significantly impairs people’s ability to work, look after their children, or engage with other activities. Despite variability in the nature and severity of reported symptoms, there are some core symptoms experienced by nearly all those with Long Covid: fatigue; cognitive impairment (“brain fog”); breathlessness; anxiety and depression (2). These core symptoms are present in numerous other long-term conditions, and treated with well established, non-pharmacological interventions, including physiotherapy, nutritional advice, cognitive behavioural approaches, sleep hygiene and improving self-management skills. These interventions are most often delivered by Allied Health Professionals (AHP), such as physiotherapists, occupational therapists, clinical psychologists, and dieticians, specialising in rehabilitation.

Prior to the Covid-19 pandemic, rehabilitation services were at capacity, and their ability to absorb over half a million additional patients requiring help has been further compromised by redeployment of many AHP to supporting acute services. Additionally, many services are system-focussed, so a patient with Long Covid who is breathless, fatigued and anxious could be directed to three different clinicians: a respiratory physiotherapist to help with breathing, a neuro-physiotherapist or occupational therapist for fatigue, and a clinical psychologist for anxiety, making treatment extremely burdensome (7).

One effect of the pandemic has been to rapidly accelerate the move toward the use of digital technologies to support health care (8). Digitally-supported rehabilitation, shown to be effective in other disease areas (9-12), has the potential to address the challenge of providing timely healthcare to large numbers of individuals with a highly constrained workforce (13). Digital health is not without its challenges: despite being shown to be effective in improving health outcomes, relatively few digital health interventions (DHI) have made it into mainstream healthcare (14, 15). There are well recognised problems with implementation at scale; many DHI suffer from poor uptake or inadequate ongoing use (16), reducing the likelihood of effectiveness. Furthermore, there are concerns around health inequalities and the digital divide, especially as Covid-19 disproportionately affects people from ethnic minority groups, socially deprived backgrounds and older people (17). People from these backgrounds are also less likely to have access to digital technology (18), so care delivered digitally risks exacerbating health inequalities. However, it has been shown that the digital divide can be overcome by careful design and implementation into routine health care (19).

Aims and objectives. The aim is to iteratively design, deploy and evaluate a digitally-mediated, remote, supported rehabilitation programme for patients affected by Covid-19, which is used effectively, deployed at scale, and does not widen health inequalities.

For peer review only - http://bmjopen.bmj.com/site/about/guidelines.xhtml

Specific objectives are to:

1. ***Develop and refine a DHI that supports patient monitoring, remote rehabilitation and identifying patients needing further specialist investigation***. To include:
   a) Clinical pathways aligned with NICE guidelines (20);
   b) A clinician-facing digital dashboard which displays data about individuals and selected cohorts;
   c) A patient-facing mobile app to provide targeted, tailored rehabilitation according to patient symptoms. The app will collect patient-reported outcome measures (PROMS) and use intelligent algorithms and machine learning to promote engagement and tailor treatment advice under clinician guidance.

2. ***Determine implementation strategies to promote adoption, scale-up, spread and sustinability with a view to maximising population impact.*** To include assessing:
   a) How best to integrate the DHI into clinical workflows, so it becomes fully normalised for healthcare professionals (HCP), and patients experience seamless care;
   b) The optimal amount of HCP input (time, skill set) required to support patients to engage effectively with the DHI, including those with low digital literacy, while managing large patient workloads safely;
   c) Funding and commissioning models to promote long-term sustainability.

3. ***Assess the population impact of this model of care.*** To include:
   a) Determining the reach (uptake and engagement) of the DHI, as a proportion of eligible patients and how this varies by demographic characteristics;
   b) Exploring its impact on patient-reported outcomes and recovery trajectories;
   c) Assessing the cost implications to the NHS of scaling-up and sustaining it.

4. ***Determine and mitigate the effect of the digital divide on health inequalities.***
   a) Identify patterns of differential uptake, use and apparent benefits of the DHI for people from ethnic minority or socially disadvantaged backgrounds;
   b) Identify and test actions to mitigate any observed differential.

Methods.

Overall Design: This project will combine research methods common to engineering and computer science (focused on developing a product that is safe, stable and meets user requirements) with those familiar to biomedical, behavioural and health service researchers (focused on effectiveness and population impact). Thus it will follow the Medical Research Council (MRC) Framework for development and evaluation of complex interventions (Phases 1, 2 and 4) (21), User-Centred Design (UCD), and the ISO 9241 Human-Computer Interaction (HCI) Lifecycle (22) for intervention development. Evaluation will use mixed methods, combining qualitative and quantitative data. The work will be divided into 4 work-packages (WP) across 2 years, with each WP addressing an objective (Figure 1).

Key principles: All work undertaken will adhere to two key principles:

a) It addresses concurrently the aims of service and research, with the intention that the two should be mutually beneficial. The service priorities are to deliver a safe, effective and cost-effective service at speed in a resource-constrained environment; research requires us to generate new, generalisable knowledge.

For peer review only - http://bmjopen.bmj.com/site/about/guidelines.xhtml

b) Inter-disciplinary approach with equal representation. The four communities represented in the team include: (i) patients, represented through Patient and Public Involvement (PPI); (ii) clinicians caring for patients with Long Covid, including Respiratory Specialists, General Practitioners, and Allied Health Professionals; (iii) industry, in the form of a small-medium enterprise (SME) specialising in digital health with substantial experience of deployment in the NHS and other healthcare systems; (iv) academics. The academic disciplines represented in the team include Computer Science, Human-Computer Interaction, Digital Health, Behavioural Science, Social Science and implementation research, Statistics, Health economics, Health services research and Clinical academics.

Patient and Public Involvement (PPI). There is strong PPI representation throughout this study. The original application was developed with members of Long Covid social media groups, and the funding application was reviewed by PPI prior to submission. A PPI member (JB) is a co-investigator, named on the funding application, sits on the project steering committee and is a co-author on this paper. Once funding was confirmed, we recruited additional PPI members resulting in 2 PPI on the steering committee, 2 PPI on each of the work package groups, and a PPI Advisory Group. PPI members have contributed to: the overall design of the protocol; conceptualisation of Long Covid, including prioritisation of the symptoms of fatigue and brain fog; writing and reviewing the content, navigation and functionality of the intervention; selecting outcome measures; data collection, particularly running focus groups with other PPI; data analysis, particularly around user requirements; and continue to contribute to the study conduct.

Timeline. This is a two year project, that started in September 2020, with WP1 mainly focused on the first year of the project to design and optimise the product. WP3 will mainly be focused on the second year. WP2 & 4 will run throughout both years.

Work Package 1. Development and refinement of the Living With Covid Recovery programme (LWCR).

Design: Inter-disciplinary team applying agile User Centred Design (UCD) and Human Computer Interaction (HCI) methods to iteratively determine user requirements, to develop and refine the DHI by testing it against user requirements including optimising effectiveness in achieving desired behavioural changes (Figure 2).

Inputs include: user requirements; emerging clinical evidence on Long Covid; evidence-based treatments; development of personas (an HCI technique involving developing rich, contextualised descriptions of a range of potential users (14)); qualitative user feedback; and quantitative usage data. These data sources will be combined in an interative fashion, with 6 development cycles planned for the first year (Figure 2), starting with an existing platform designed for use in other chronic health conditions such as cancer, rhumatoid arthritis and incontinence.

Participants: We have identified three main stakeholder groups for user requirements: patients with Long Covid; Health Care Professionals (HCP) caring for patients with Long Covid; and health service managers responsible for Long Covid services. Initally, patient user requirements have been determined from PPI; recent ethics approval will allow data collection from patients using the LWCR programme. HCP and health service managers wil be identified initially through snowballing from the research team and subsequently from NHS teams using the service.

Data collection: We will use qualitative methods in real-time, including online meetings, interviews and focus groups with the three stakeholder groups, recorded with participant consent. Meetings

For peer review only - http://bmjopen.bmj.com/site/about/guidelines.xhtml

will be driven by both research and service need to provide real-time insight into the needs of patients, health service managers and HCP, as they adapt to the evolving pandemic, without imposing additional workload. Documents, including meeting agendas and minutes, and follow-up emails, will also be used as data sources.

Quantitative data: see WP3. For ethical reasons we are unable to contact or collect data directly from patients who have declined the LWCR service.

Data analysis: Recordings will be stored securely. Relevant sections will be transcribed by a General Data Protection Regulation (GDPR) compliant transcribing company, anonymised, and checked for accuracy. Analysis will be done in two stages: first, a rapid, emergent analysis in parallel with data collection, focusing on actionable findings, fed immediately into the development process. Different data sources will be synthesised by the inter-disciplinary development team, through iterative discussions. Subsequent analysis will be in depth and inductive with transcripts coded by experienced qualitative researchers using NVivo, with additional deductive analysis where appropriate, driven by one of our theoretical frameworks or findings from the literature, critiqued in a multi-disciplinary data clinic.

Quantitative data will be anonymised. Initial analyses will be descriptive, with subsequent, more complex analyses focusing on usage patterns with a view to maximising uptake and use.

Outputs:

Evidence-based DHI which is rapidly and fully integrated into routine care pathways of NHS Long Covid clinics nationally. The DHI will be routinely updated with new features for the App and Dashboard such as questionnaries or content every few months to increase usabiliy, engagement and effectiveness based on participant feedback. Patients and Clinicians will be made aware of changes through notifications on the DHI or emails and supported with a helpline.

Work Package 2: Determine implementation strategies to promote adoption, scale-up, spread and sustainability with a view to maximising population impact.

Design

Mixed methods, using quantitative data to measure uptake and use at individual and clinic level, and qualitative data to explore and understand barriers and facilitators to implementation. Data collection, analysis and development of implementation strategies will be informed by Normalisation Process Theory (NPT) (15) (23), a sociological theory concerned with the work required to implement, embed and integrate (or “normalise”) new practices or technologies into routine healthcare.

Setting

The LWCR programme will be implemented in community Long Covid clinics nationally; these clinics will cover both urban and rural areas. The use of the LWCR programme is free for any NHS long covid clinic in England until September 2022. We conceptualise implementation as occuring at the macro, meso and micro level. We define macro as system level change, such as change across multiple local health economies at national or regional level, for example NHS England (NHSE), Academic Health Science Networks (AHSNs) (24) or Integrated Care Systems (ICS) (25). Meso refers to change across a single clinic or local health economy; and micro refers to change at the level of the individual clinician or patient.

For peer review only - http://bmjopen.bmj.com/site/about/guidelines.xhtml

Participants

Participants for this WP include anyone with responsibility for implementing change in the delivery of care for patients with Long Covid at any level. This includes staff working for NHSE or the Department of Health and Social Care (DHSC) with responsibility for policy and practice; commissioners of care; local health service leaders, such as leads or staff working for AHSNs or ICS, clinical and managerial leads working within or across health service Trusts (acute, primary or community care), clinicians responsible for specific clinics and / or delivering front line care to patients with Long Covid, and patients with Long Covid or their carers. Participants also include professionals with particular relevant expertise needed to support the implementation of LWCR, such as understanding of Information Governance or procurement regulations in the NHS, and members of the research team with responsibility for setting up and delivering the service.

Data collection

Data collection and analysis will be shared across WPs. Additional quantitative data collected for WP2 includes numbers of Trusts showing initial interest in deployment who decide against adoption, and demographic data on HCP registered on the dashboard, including clinical specialty, level of experience, age, gender and ethnicity, collected through a proforma questionnaire completed by each clinic once only.

Additional qualitative data will include email trails and documents published by DHSC, NHSE, AHSNs, ICS or other health service bodies. Clinician feedback on the service will be collected as part of the cycles of iterative development and refinement. This service-related data may be augmented, where necessary, by specific interviews with selected invidivuals, for example to obtain deeper insights into an issue raised in a meeting, or to test emerging interpretations of data. Where possible meetings will be recorded, with participant consent. Field notes and minutes will also form part of the dataset.

Data pertaining to individual uptake and use of the LWCR programme will include feedback from clinicians, particularly focusing on how they introduce the service to patients and steps taken to encourage uptake and use, particularly amongst patients who are less used to digital technology. Opt-in consent will be sought from patients and clinicians to record specific on-boarding consultations. Patients will be invited to participate in interviews with the research team to explore their experience of Long Covid, the extent to which the service meets their perceived needs, and what could be done to improve the service for others.

Data analysis

Analysis of quantitative data is described in WP3. Qualitative data will be analysed in two stages, as described for WP1, with the first stage focusing on actionable findings to promote deployment and the second focusing on transferable learning about implementation of digital health interventions. In this second stage, data will be analysed inductively, with data-driven codes subsequently mapped onto NPT concepts, looking specifically for disconfirming data or data that does not map onto NPT.

Outputs from WP2 will include:

For peer review only - http://bmjopen.bmj.com/site/about/guidelines.xhtml

i) a fully developed implementation package, detailing the process of successful implementation of a digital health intervention into routine health care in a range of healthcare setting (primary, secondary and community care);
ii) a sustainable, scalable business model, allowing for ongoing maintenance and development of the product while it is used at scale;
iii) transferable learning about optimising the integration of digital health into routine care, ensuring optimal use by healthcare professionals and patients, including an understanding of how implementation models can mitigate the digital divide.

Work Package 3: Assess the population impact of this model of care.

Design: Quantitative workpackage. Population impact is defined as reach multiplied by effect.

Participants: The target for year 1 will be to recruit 1,000 patients registering with the DHI. This will enable us to estimate the overall proportion of patients engaging with the app with a high-level of precision (maximum width of 95% confidence interval is +/- 3.2%). Similar or increased levels of precision will be achieved when estimating the proportion of those referred who register with the intervention. The sample size in year 2 has not been pre-specified and will depend upon practical constraints imposed by the scale of the pandemic as the digital intervention is rolled out nationally. Power calculations will be carried out for the year 2 analyses based on the 'pilot' data from year 1. This will include power for the proposed quasi-experimental analysis of the effect of the DHI on outcomes at 3- and 6-months follow-up.

Data collection:
Quantitative data on numbers of patients, healthcare professionals and Long Covid clinics registered to use LWCR. Additionally, patient demographic data clinical and service data and app usage patterns, as recorded through the LWCR platform will be provided.

Demographic data will be obtained on patients' age, gender, ethnicity, highest level of educational attainment and socio-economic status (using Index of Multiple Deprivation, calculated from the postcode) when patients first register on the app. This data will be entered by healthcare professionals or the patients themselves.

Clinical data will be obtained from patient-completed Patient Reported Outcome Measures (PROMS) on the app. Patients will be asked to complete all the PROMS at baseline to help with clinical assessment, and thereafter, only those PROMS which the clinician and patient deem to be clinically relevant and helpful for clinical management.

Validated PROMS include: the Work and Social Adjustment Scale (WSAS) which measures functional impairment and is our primary outcome measure (26); EQ-5D-5L for health related quality of life (27); FACIT-F for fatigue (28); GAD-7 for anxiety (29); PHQ-8 for depression (30); MRC dyspnoea scale (22) and Dyspnoea-12 (31) for breathlessness; and PDQ-5 for cognitive impairment (32). Additional questions explore levels of physical activity and overall recovery from Covid-19.

Service use data will be obtained when patients complete a service use questionnaire (every 4 weeks), detailing the number of healthcare appointments, days off work and days as a hospital inpatient they have experienced.

Usage data, including the date and time of each page view by each patient are recorded automatically by the programme.

For peer review only - http://bmjopen.bmj.com/site/about/guidelines.xhtml

Data analysis

Each patient will be assigned a unique participant identification number (PIN). The linking key will be held by Living With, as data processors for the Clinical Trusts. Pseudonymised data, where date of birth is replaced by age and postcode replaced with IMD, will be shared with the research team. Demographic characteristics and baseline symptoms of the registered 'treatment seeking' population of long COVID sufferers will be profiled using descriptive statistics.

Usage and engagement. Patient engagement with the app will be reported using a stratification of patients into low, medium and high categories based on numbher of log ins over the first 12 weeks. Demographic and baseline clinical factors influencing uptake and use will be identified through logistic regression, with multivariate methods used to model patterns of app engagement over time.

Recovery trajectories. Generalised linear mixed models (GLMM) and latent class trajectory modelling (LCTM) will be used to examine stability and change in recovery trajectories for the longitudinal PROMS data, and to identify homogeneous clusters of patients with contrasting clincal phenotypes. Time since registration with the programme will initially be included as a linear covariate, but we will explore the functional form of the effect of time to allow for phenomena such as a plateau in symptom recovery or a period of sudden rapid improvement or relapse. Models will include random slopes and intercepts, allowing for individualised differences in severity of baseline symptoms and subsequent trajectories. Fixed effects will include age, gender, ethnicity, IMD and educational attainment.

Mediation analysis of intervention components. A mediation analysis will be undertaken in which intervention components and measures of patient app usage and engagement (number of log ins and assessment completed) will be added to longitudinal models as explanatory factors. We will explore whether increased engagment by clinicians such as messages sent and reviews of patient assessments on the dashboard is linked with improved patient outcomes. We will assess whether there is a dose-response relationship between usage of the app / introduction of new content and change in outcomes.

Effectiveness of the LWCR programme. We will investigate the feasibility of creating a synthetic comparator arm from patients with long Covid enrolled in suitable comparator cohorts, hosted in BREATHE-SAIL (33). Direct comparisons will be made between patients receiving the evidence-based interventions and the comparator group(s) in multivariable GLMMs with propensity score methods used to provide control for measured confounders.

Equality. The acceptability of the intervention for people from disadvantaged backgrounds will be explored through logistic regression analysis of these factors as predictors of recruitment, usage and retention. Differential effects of the DHI on outcomes for disadvantaged groups will be investigated through inclusion of interactions between intervention uptake and ethnicity, age, educational status, and IMD in the GLMMs.

Missing data. Sensitivity analyses will be conducted to examine the influence of missing data on the key study findings. This will include the use of multiple imputation methods, where the assumption that the data are missing at random (MAR) is considered appropriate, and tipping point analysis (34) to assess sensitivity of the results to data that are missing not at random (MNAR).

Health economic evaluation.

For peer review only - http://bmjopen.bmj.com/site/about/guidelines.xhtml

The health economic evaluation will estimate the cost of delivering the DHI to the target population, the cost consequences of the DHI for the NHS services compared to standard care, and a budget impact analysis over the next 3-5 years (35) that assesses the costs and potential savings resulting from commissioning the proposed DHI. To generate costs from the resource use data, appropriate unit costs; e.g. price lists from the app developer to cost items related to app development, and Unit Costs for Health and Social Care (36) to cost staff time, will be applied.

Relevant costs will be identified by examining how the DHI impacts the patient, hospital, and the health care system. These will include:

- Resources associated with the development and hosting of the patient-facing app.
- Resources incurred with the scaling up the DHI, such as maintenance, updating, data storage, implementation, and training.
- Resource use associated with HCP support of patients using the service.
- Impact on health service use.

Outputs.

Outputs from WP3 will include detailed understanding of patient symptoms and illness trajectories over time, estimates of the impact of the digital health programme on these trajectories and an understanding of the relative cost-effectiveness of the LWCR programme compared with standard care i.e. the care provided to those patients not supported by the LWCR programme.

Work Package 4: Determine and mitigate the effect of the digital divide on health inequalities.

Design. This cross-cutting WP will ensure that the work undertaken in the other three WPs is fully sensitive to the needs of those who might be at risk of exclusion, such as minority ethnic groups, socially disadvantaged or older people. It will seek to identify evidence of the digital divide and health inequalities, and work to mitigate these. We will undertake a systematic scoping review to identify features of DHI design and deployment that enable engagement by people with low digital/health literacy, e.g. key content is presented using graphics, video, and audio to enhance accessibility.

In relation to WP1 the focus will be on ensuring that the personas used in developmental design include minority ethnic, socially disadvantaged, and older people, to improve usability and engagement in these groups. We will ensure the content is written for a reading age of entry level 3 (attained by over 80% of the UK population) (37) and therefore understandable by most patients. Design features will allow family or carers to log in and support patients. The systematic scoping review will inform intervention design and deployment to maximise engagement by people with low digital/health literacy.

In relation to WP2 the focus will be on identifying and exploring factors in the implementation process that could hinder uptake and increase health inequalities, or conversely, help to mitigate inequalities. For example, translation of key content into different languages, the use of digital champions to help support patients, referral to charities that supply smart phones and advise how to access low-cost data or free Wi-Fi to help facilitate increased access to all groups.

In relation to WP3 the focus will be on ensuring the analysis of the outcome measures can be compared across different demographic groups so that disparities can be identified. Exploring the factors that can influence differential uptake, use, and impact across the different clinics and demographic groups.

For peer review only - http://bmjopen.bmj.com/site/about/guidelines.xhtml

Outputs. An intervention which is accessible to people from a range of demographic backgrounds and that collects/analyses/acts on data suggesting differential uptake or impact of the digital health intervention by people from these demographic groups; and an understanding of the features of the implementation model which promote participation by people from ethnic minority, disadvantaged, or older groups.

Ethics and dissemination

This protocol was approved by the East Midlands – Derby Research Ethics Committee (reference 288199). Ethical considerations was given to obtaining patient consent and maintaining patient anonimity. First consent to contact patients by email was obtained using a tick box on the App. Only those patients that opted in will be contacted and ask if they wish to participate in interviews. All patient data collected from the LWCR product was psudeo-anonymised by Living With. Patient email addresses from those who agreed to be contacted will be stored separately in the university data safe haven in accordance with the GDPR and data goverance protocols.

Our dissemination strategy targets three audiences: (i) Policy makers, Health service managers and clinicians responsible for delivering Long Covid services; (ii) Patients and the public; (iii) Academics. Findings will be disseminated at conferences and papers to target academics, through nationally advertised workshops for clinicians and administrators working in or running long covid clinics, and through news and social media for patients.

Discussion.

This protocol describes the processes for developing, deploying and evaluating digitally-enabled remote, supported rehabilitation for people with Long Covid syndrome. Strengths include the inter-disciplinary approach, with a partnership between patients, clinicians, industry and academics, plus combining approaches used in engineering / computer science, e.g. UCD and the HCI lifecycle, with those more familiar to biomedical researchers, e.g. the MRC Framework for complex interventions. The strong use of theory to underpin the research should enhance transferability of findings. Putting equal priority on service needs and research is innovative, but brings challenges. These include relying on diversity of service delivery for obtaining an appropriate comparator and the reliance on data obtained for clinical purposes for the impact evaluation. We expect there to be substantial amounts of missing data, particularly in the PROMS, as clinicians and patients are likely to focus on recording symptoms of most interest to them or suffering from questionnaire fatigue. However, no one study can answer all relevant research questions about an innovation, and we agree with the argument that a digital health intervention should achieve a reasonable level of stability before being evaluated in a randomised controlled trial (38).. This initial study will provide considerable insight into patient, clinician and healthservice requirements for digitally-supported rehabilitation for Long Covid, together with very substantial, real world data on acceptability, uptake and use, going far beyond the usual pilot studies so often used as evidence of acceptability and feasibility, with data on scalable, and hopefully, sustainable, deployment. It will also generate data on symptoms and illness trajectories in a treatment-seeking population which includes non-hospitalised patients, making it unique amongst current cohort studies. Long term investment will require causal evidence of effectiveness, and NIHR have funded the STIMULATE-ICP project, which includes a cluster randomised controlled trial comparing LWCR to standard care (39), due to report in 2023. Other future work will consider whether a digital tool could help stratify Long Covid patietns by their

For peer review only - http://bmjopen.bmj.com/site/about/guidelines.xhtml

clinical severity (40) or combine with other digital help products such as telemedicine to achieve better outcomes (41).

We believe that our approach could help alleviate the well known delay in translating research findings into practice (42) whilst simultaneously promoting the sustainable, scalable adoption of evidence-based interventions and adding to the research base in digital health.

For peer review only

For peer review only - http://bmjopen.bmj.com/site/about/guidelines.xhtml

For peer review only

For peer review only - http://bmjopen.bmj.com/site/about/guidelines.xhtml

Author Contributions.

All authors (EM, HG, JB, AB, KB, DFR, MG, FH, MH, WH, JH, HH, SL, PP, WR, CR, RS, FS and JW), with the exception of TC and SW, contributed to the development of the successful funding application on which this protocol is based. TC further developed and operationalised the protocol as part of the ethics submission and SW elaborated the statistical analyses. EM and HG are co-chief investigators and JB, AB, KB, DFR, MG, FH, MH, WH, JH, HH, SL, PP, WR, CR, RS, FS and JW are co-investigators on the project. EM wrote the first draft of the paper and incorporated comments on the draft manuscript from all authors. HG wrote the response to the reviewer comments after discussion and comments from all authors. All authors have read and approved the final version.

Funding statement.

This study is funded by the National Institute for Health Research (NIHR) Crossprogramme [HS&DR] COVID-19 [project reference NIHR132243 - Supported remote rehabilitation post Covid-19: development, deployment and evaluation of a digitally-enabled rehabilitation programme]. The views expressed are those of the author(s) and not necessarily those of the NIHR or the Department of Health and Social Care.
This report is independent research supported by the National Institute for Health Research ARC North Thames.

Competing interests statement.

Chris Robson and Jonathan Waywell are directors of the provider company, *Living With*, which has a suite of digital health interventions used in healthcare systems including the NHS.

Data Statement

Data generated by this study will be deposited with the Breathe-Sail database.

Word Count: 3,953

References

1. Guan WJ, Ni ZY, Hu Y, Liang WH, Ou CQ, He JX, et al. Clinical Characteristics of Coronavirus Disease 2019 in China. The New England journal of medicine. 2020;382(18):1708-20.
2. Crook H, Raza S, Nowell J, Young M, Edison P. Long covid—mechanisms, risk factors, and management. BMJ (Clinical research ed). 2021;374:n1648.
3. Castaranes-Zapatero DH, G; van den Heede, K; . Epidemiology of long COVID: a pragmatic review of the literature. Brussels: KCE; 2021 27/01/21.
4. NIHR. Living With Covid19. A dynamic review of the evidence around ongoing Covid19 (often called Long Covid). London; 2021 16/03/2021.
5. Mandal S, Barnett J, Brill SE, Brown JS, Denneny EK, Hare SS, et al. 'Long-COVID': a cross-sectional study of persisting symptoms, biomarker and imaging abnormalities following hospitalisation for COVID-19. Thorax. 2021;76(4):396-8.
6. Taylor RR, Trivedi B, Patel N, Singh R, Ricketts WM, Elliott K, et al. Post-COVID symptoms reported at asynchronous virtual review and stratified follow-up after COVID-19 pneumonia. Clinical medicine (London, England). 2021;21(4):e384-91.

For peer review only - http://bmjopen.bmj.com/site/about/guidelines.xhtml

7. May CR, Eton DT, Boehmer K, Gallacher K, Hunt K, MacDonald S, et al. Rethinking the patient: using Burden of Treatment Theory to understand the changing dynamics of illness. BMC health services research. 2014;14:281.
8. Hutchings R. The impact of Covid-19 on the use of digital technology in the NHS. Briefing August 2020. London; 2020.
9. Dunphy E, Button K, Hamilton F, Williams J, Spasic I, Murray E. Feasibility randomised controlled trial comparing TRAK-ACL digital rehabilitation intervention plus treatment as usual versus treatment as usual for patients following anterior cruciate ligament reconstruction. BMJ open sport & exercise medicine. 2021;7(2):e001002.
10. Geraghty AWA, Stanford R, Stuart B, Little P, Roberts LC, Foster NE, et al. Using an internet intervention to support self-management of low back pain in primary care: findings from a randomised controlled feasibility trial (SupportBack). BMJ open. 2018;8(3):e016768.
11. Bourne S, DeVos R, North M, Chauhan A, Green B, Brown T, et al. Online versus face-to-face pulmonary rehabilitation for patients with chronic obstructive pulmonary disease: randomised controlled trial. BMJ open. 2017;7(7):e014580.
12. Widmer RJ, Allison TG, Lennon R, Lopez-Jimenez F, Lerman LO, Lerman A. Digital health intervention during cardiac rehabilitation: A randomized controlled trial. Am Heart J. 2017;188:65-72.
13. Providers N. SPOTLIGHT ON... DIGITAL TRANSFORMATION IN RESPONSE TO COVID-19. 2020.
14. Greenhalgh T, Wherton J, Papoutsi C, Lynch J, Hughes G, A'Court C, et al. Beyond Adoption: A New Framework for Theorizing and Evaluating Nonadoption, Abandonment, and Challenges to the Scale-Up, Spread, and Sustainability of Health and Care Technologies. Journal of medical Internet research. 2017;19(11):e367.
15. Ross J, Stevenson F, Lau R, Murray E. Factors that influence the implementation of e-Helath: A systematic review of systematic reviews (an update). Implementation science : IS. 2016.
16. Perski O, Blandford A, West R, Michie S. Conceptualising engagement with digital behaviour change interventions: a systematic review using principles from critical interpretive synthesis. Translational behavioral medicine. 2017;7(2):254-67.
17. Patel JA, Nielsen FBH, Badiani AA, Assi S, Unadkat VA, Patel B, et al. Poverty, inequality and COVID-19: the forgotten vulnerable. Public Health 2020;183:110-1.
18. Powell CB, L. Digital Divide: Mapping the geodemographics of internet accessibility across Great Britain: arXiv; 2021 [Available from: https://arxiv.org/abs/2108.07699.
19. Poduval S, Ahmed S, Marston L, Hamilton F, Murray E. Crossing the Digital Divide in Online Self-Management Support: Analysis of Usage Data From HeLP-Diabetes. JMIR Diabetes. 2018;3(4):e10925.
20. NICE. Covid-19 Rapid Guideline: Managing Covid-19. London; 2021 10/08/21.
21. Craig P, Dieppe P, Macintyre S, Michie S, Nazareth I, Petticrew M. Developing and evaluating complex interventions: the new Medical Research Council guidance. BMJ. 2008;337:a1655. doi: 10.1136/bmj.a1655.:a1655.
22. Sharp H, Preece J, Rogers Y. Interaction Design: Beyond Human-Computer Interaction. 5th ed: Wiley; 2019.
23. May C, Finch T. Implementation, embedding, and integration: an outline of Normalization Process Theory. Sociology. 2009;43(3):535-54.
24. Network TA. Academic Health Science Networks: Transforming lives through healthcare innovation 2021 [Available from: https://www.ahsnnetwork.com/.
25. England N. What is Integrated Care? 2021 [Available from: https://www.england.nhs.uk/integratedcare/what-is-integrated-care/.
26. Mundt JC, Marks IM, Shear MK, Greist JH. The Work and Social Adjustment Scale: a simple measure of impairment in functioning. The British journal of psychiatry : the journal of mental science. 2002;180:461-4.

For peer review only - http://bmjopen.bmj.com/site/about/guidelines.xhtml

27. Herdman M, Gudex C, Lloyd A, Janssen M, Kind P, Parkin D, et al. Development and preliminary testing of the new five-level version of EQ-5D (EQ-5D-5L). Quality of life research : an international journal of quality of life aspects of treatment, care and rehabilitation. 2011;20(10):1727-36.
28. Lai JS, Cella D, Choi S, Junghaenel DU, Christodoulou C, Gershon R, et al. How item banks and their application can influence measurement practice in rehabilitation medicine: a PROMIS fatigue item bank example. Archives of physical medicine and rehabilitation. 2011;92(10 Suppl):S20-7.
29. Spitzer RL, Kroenke K, Williams JB, Löwe B. A brief measure for assessing generalized anxiety disorder: the GAD-7. Arch Intern Med. 2006;166.
30. Kroenke K, Strine TW, Spitzer RL, Williams JBW, Berry JT, Mokdad AH. The PHQ-8 as a measure of current depression in the general population. J Affective Disorders. 2009;114.
31. Yorke J, Swigris J, Russell AM, Moosavi SH, Ng Man Kwong G, Longshaw M, et al. Dyspnea-12 is a valid and reliable measure of breathlessness in patients with interstitial lung disease. Chest. 2011;139(1):159-64.
32. Julian LJ, Yazdany J, Trupin L, Criswell LA, Yelin E, Katz PP. Validity of brief screening tools for cognitive impairment in rheumatoid arthritis and systemic lupus erythematosus. Arthritis care & research. 2012;64(3):448-54.
33. UK HDR. Breathe: Our Hub for Respiratory Health 2021 [Available from: https://www.hdruk.ac.uk/helping-with-health-data/our-hubs-across-the-uk/breathe/.
34. Yan X, Lee S, Li N. Missing data handling methods in medical device clinical trials. Journal of biopharmaceutical statistics. 2009;19(6):1085-98.
35. Assessing resource impact manual. London; 2021.
36. Curtis LB, A. . Unit Costs of Health and Social Care 2020. Canterbury: Personal Social Services Research Unit, University of Kent; 2020.
37. Trust L. What do adult literacy levels mean? 2021 [Available from: https://literacytrust.org.uk/parents-and-families/adult-literacy/what-do-adult-literacy-levels-mean/), .
38. Murray E, Hekler EB, Andersson G, Collins LM, Doherty A, Hollis C, et al. Evaluating Digital Health Interventions: Key Questions and Approaches. American journal of preventive medicine. 2016;51(5):843-51.
39. New research into treatment and diagnosis of long COVID [press release]. 18.07.2021 2021.
40. Yan, Z.; Yang, M.; Lai, C.-L., Long COVID-19 Syndrome: A Comprehensive Review of Its Effect on Various Organ Systems and Recommendation on Rehabilitation Plans. Biomedicines 2021, 9 (8), 966
41. Kichloo A, Albosta M, Dettloff K, Wani F, El-Amir Z, Singh J, et al. Telemedicine, the current COVID-19 pandemic and the future: a narrative review and perspectives moving forward in the USA. Fam Med Community Health. 2020;8(3).
42. Grol R, Grimshaw J. From best evidence to best practice: effective implementation of change in patients' care. Lancet. 2003;362(9391):1225-30.

For peer review only

Figure Legend:

Figure 1: Living With Covid Recovery Organisational Structure

Figure 2: Iterative cycles of development and refinement for Living With Covid Recovery

For peer review only

For peer review only - http://bmjopen.bmj.com/site/about/guidelines.xhtml

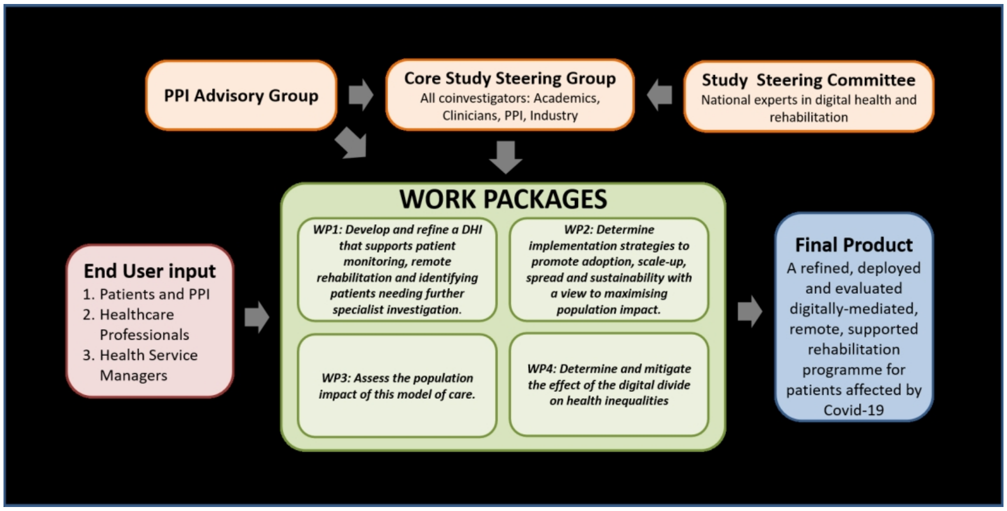

Figure 1 Living With Covid Recovery Organisational Structure

146x73mm (300 x 300 DPI)

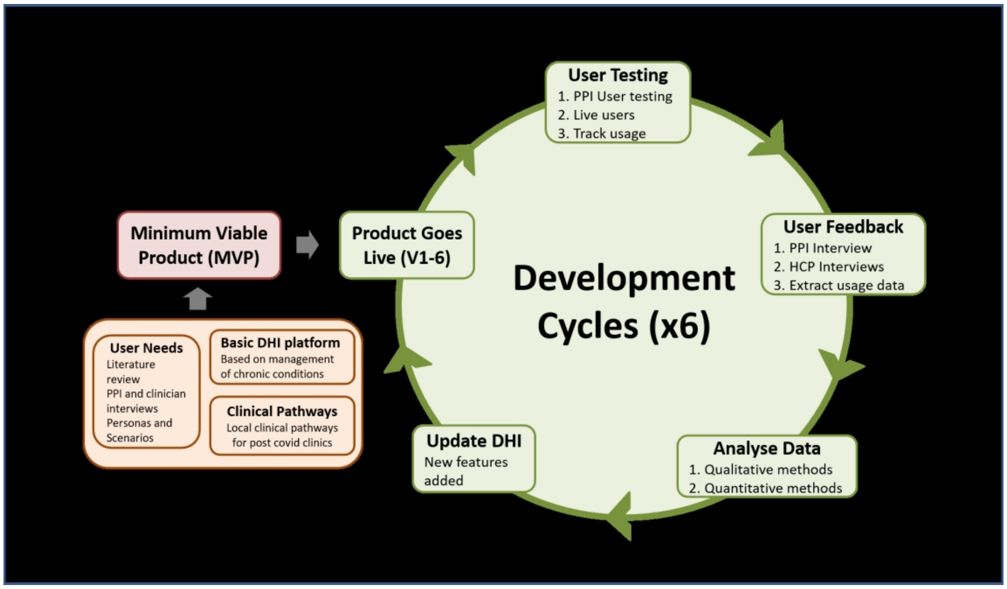

Figure 2 Iterative cycles of development and refinement for Living With Covid Recovery

159x92mm (300 x 300 DPI)
